# Supplementary material for: EXSCLAIM!: Harnessing materials science literature for self-labeled microscopy datasets
Source: Patterns (N Y). 2023 Sep 30;4(11):100843. doi: 10.1016/j.patter.2023.100843 (PMC10682750; doi:10.1016/j.patter.2023.100843)
Supplement: Document S1. Supplemental notes, Figures S1–S3, and supplemental experimental procedures [file mmc1.pdf]

Patterns, Volume *4*

Supplemental information

EXSCLAIM!: Harnessing materials science literature for self-labeled microscopy datasets

Eric Schwenker, Weixin Jiang, Trevor Spreadbury, Nicola Ferrier, Oliver Cossairt, and Maria K.Y. Chan

Supplemental Notes

Introduction

The sections outlined in this document provide further details on results and experimental procedures, in support of the conclusions made in the corresponding sections of the main paper.

Results

Journal Scraper

The reason that the default parsers are designed for Nature, ACS, and RSC families relates to both impact and the fact that their contents are served in fundamentally different ways. Nature and ACS have static content, while RSC's content is served dynamically. Consequently, with this collection of parsers, we cover two common ways information could be obtained from a website, which leaves this tool open to be easily extended to any open source journal. All figure/caption extraction is performed directly from the HTML article and does not require PDF downloads. This is a design choice based on the observation that most open-source content is available directly from the HTML text and the ability to download the PDF is a convenience rather than a necessity.

Figure Separator

The *figure separator* comprises two primary modules:

1. Subfigure Label Detector (YOLOv3 + ResNet152)

   YOLOv3 is trained for label detection and ResNet152 is trained for label recognition. For the YOLOv3 implementation, refer to: https://github.com/WeixinGithubJiang/PyTorch_YOLOv3

   YOLOv3:

```
MODEL:
  TYPE: YOLOv3
  BACKBONE: darknet53
  ANCHORS: [[6, 7], [9, 10], [10, 14],
            [13, 11], [16, 15], [15, 20],
            [21, 19], [24, 24], [34, 31]]
  ANCH_MASK: [[6, 7, 8], [3, 4, 5], [0, 1, 2]]
  N_CLASSES: 15
TRAIN:
  LR: 0.001
  MOMENTUM: 0.9
  DECAY: 0.0005
  BURN_IN: 1000
  MAXITER: 20000
  STEPS: (400000, 450000)
  BATCHSIZE: 4
  SUBDIVISION: 16
  IMGSIZE: 608
  LOSSTYPE: l2
  IGNORETHRE: 0.7
AUGMENTATION:
  RANDRESIZE: True
  JITTER: 0.3
  RANDOM_PLACING: True
  HUE: 0.1
  SATURATION: 1.5
  EXPOSURE: 1.5
  LRFLIP: False
  RANDOM_DISTORT: True
TEST:
  CONFTHRE: 0.01
  NMSTHRE: 0.1
  IMGSIZE: 608
NUM_GPUS: 1
GPU_ID: 0
```

   ResNet152:

   We used a pre-trained model from ImageNet as weight initialization, then trained with a mixture of real MTurk-labeled images and synthetic. The Adam optimizer was used with initial training weights 0.001 and decay every 10000 iteration. A seed was not used.

2. Subfigure detector (YOLOv3)

Model details are similar to subfigure label detection, however, the config is a bit different (KMeans was used to find the proper anchor boxes).

YOLOv3:

```
MODEL:
  TYPE: YOLOv3
  BACKBONE: darknet53
  ANCHORS: [[185, 147], [94, 87], [133, 126],
            [102, 212], [234, 102], [204, 199],
            [390, 123], [177, 368], [318, 230]]
  ANCH_MASK: [[6, 7, 8], [3, 4, 5], [0, 1, 2]]
  N_CLASSES: 15
TRAIN:
  LR: 0.001
  MOMENTUM: 0.9
  DECAY: 0.0005
  BURN_IN: 1000
  MAXITER: 50000
  STEPS: (400000, 450000)
  BATCHSIZE: 4
  SUBDIVISION: 16
  IMGSIZE: 608
  LOSSTYPE: l2
  IGNORETHRE: 0.7
AUGMENTATION:
  RANDRESIZE: True
  JITTER: 0.3
  RANDOM_PLACING: True
  HUE: 0.1
  SATURATION: 1.5
  EXPOSURE: 1.5
  LRFLIP: False
  RANDOM_DISTORT: True
TEST:
  CONFTHRE: 0.1
  NMSTHRE: 0.1
  IMGSIZE: 416
NUM_GPUS: 1
GPU_ID: 0
```

Both YOLOv3 models were trained on ≈800 figures with SGD. Testing comprised 1164 images without a dedicated validation set (some further details are included in the ICIP paper https://arxiv.org/pdf/2101.09903.pdf). For ResNet152, training is on-the-fly because the synthesized images are re-generated every epoch. All models are trained on 2 TITAN-X GPUs, each one with 12GB memory.

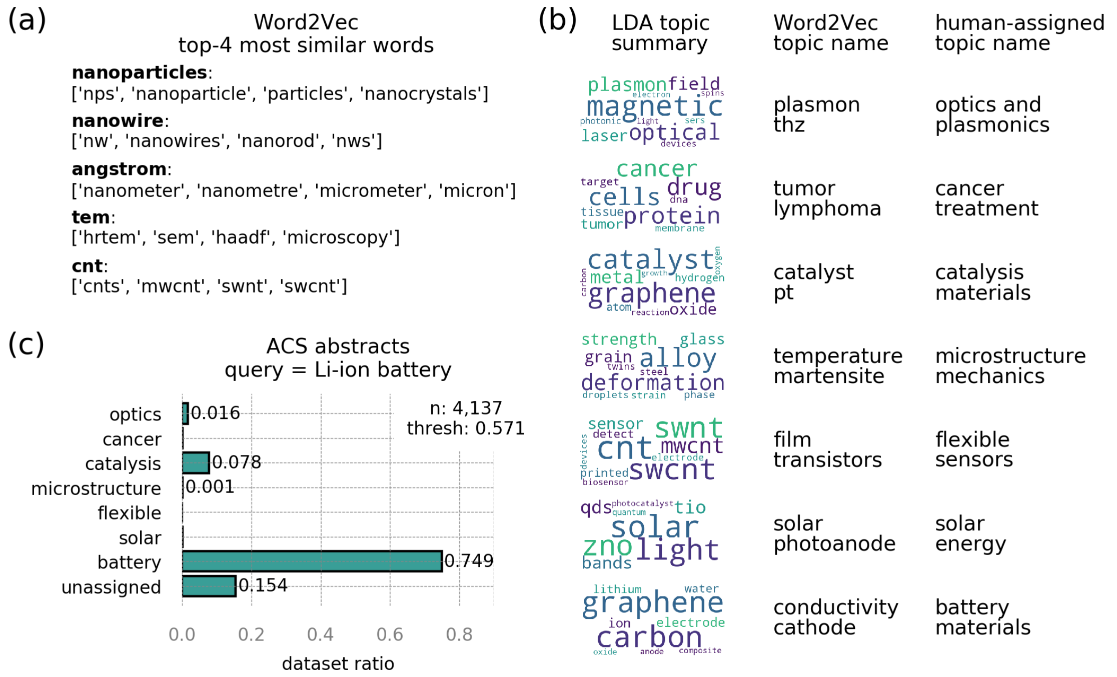

Figure S1. **Word Embeddings and Topic Modeling Highlights.** (a) Word embedding examples for a Word2Vec model trained on abstract and introduction texts from the nanostructure query. (b) LDA topic modeling applied to the introduction and abstract texts reveals some of the most popular technological applications of nanostructures. The Word2Vec topic name and human-assigned topic name represent further attempts to summarize the words of the topics into more concise titles. (c) Distribution of topics assigned to a group of 4237 abstracts collected from a query of American Chemical Society (ACS) journals for Li-ion batteries.

Annotation Expansion with NLP

The approach for determining topics and "semantic word similarity" in the main paper, uses two popular techniques from NLP: word embeddings and statistical topic modeling. These concepts are further explained below.

1. Word Embeddings (Word2Vec)

EXSCLAIM! leverages the popular unsupervised Word2Vec technique[1] to learn high quality vectors (embeddings) for words associated with the captions returned in the nanostructure query. To demonstrate how Word2Vec can be used for word associations, Figure S1a highlights simple "closest words" lookup examples. Without being explicitly associated, "nanoparticles" and "nanowire" are placed in close proximity to their abbreviations, "nps" and "nw", respectively. Additionally, 3D "nanoparticles" are closely associated with another 3D nanostructure, such as a "nanocrystal", and "nanowire" is placed near a similar 2D "nanorod". The "angstrom" unit of length is placed closely to "nanometer" and "micrometer", and interestingly, scale is even preserved in the ordering (*i.e.,* angstrom is closer in scale to a nm than μm). These sorts of quantitative relationships are not uncommon and are described in further detail in the original paper.[1] The `gensim` Python library (https://radimrehurek.com/gensim/) was used to train a Word2Vec model on 26k abstract and introduction texts scraped from nanoscience journals, with the following parameters: `min_count = 25, size = 200, iter = 500`.

2. Statistical Topic Modeling (Latent Dirichlet Analysis)

When all abstract and introductory texts related to the general nanostructures *search query* are considered together, certain topics (groups of related words) arise from high-frequency word occurrences and common word orderings. The word clouds in Figure S1b illustrate how Latent Dirichlet Analysis (LDA),[2] a popular technique used for topic modeling, provides an unsupervised clustering of related words, (from the nanostructure query) into topics. Unfortunately, LDA does not provide a topic name to the words it clusters together. To this end, we illustrate how Word2Vec can be leveraged to create topic names, and show how they align with rational human-suggested titles. For example, the topic containing "catalyst", "metal", "graphene", "reaction", etc. is given the Word2Vec topic name of "catalyst", "pt", which is easily understood to represent the general class of "catalysis materials". To demonstrate that the LDA has learned topics in an appropriate fashion, we collected 4137 abstract and introduction texts from a *search query* of ACS journal family for "Li-ion batteries", and in Figure S1c, we observe that the majority of the documents were categorized explicitly as belonging to batteries, or the highly related/overlapping catalysis category.

For LDA topic modeling, abstract and introduction texts were transformed into a corpus of TFIDF vectors and LdaMulticore was used for training with `num_topics=7 ,passes=64 ,workers=4`. The number of topics was selected based on its coherence score (`u_mass`). The coherence score can be used to assess the quality of the learned topics for a given number of possible topics. While the specific intent of the original paper describing the `u_mass` evaluation metric[3] was to develop methods to create large numbers of high-quality topics from domain-specific corpora, for purposes of demonstrating the potential utility of unsupervised topic models in context of the EXSCLAIM! pipeline, we restrict our analysis of topic coherence to a relatively small number of topics (5 - 10). This allows the reader to make a simple quality judgment, and is a generally accepted approach (*i.e.,* including a small number biases selection against the potential for *very poor* topic selection). For the specific `u_mass` metric, numbers closer to zero indicate higher coherence, as the measure itself is the log ratio of a document frequency of specific word type pairs to a document frequency of a specific word type.[3] The following table shows each topic number with its corresponding `u_mass` value, and reveals in this case that the choice of "7" topics has the best coherence score.

Topics	Coherence (`u_mass`)
5	-3.143
6	-4.354
**7**	**-1.857**
8	-4.792
9	-5.429
10	-6.122

Experimental Procedures

Custom Regex Matching

(a) **Caption Text (Raw)** Text adapted from: *Inorg. Chem. Front.*, 2019, **6**, 1382-1387

(a) **and** **(b)** TEM images of 1.93 wt% Ru–WSe2. **(c)** HRTEM image of 1.93 wt% Ru–WSe2. **(d, e)** The enlarged area denoted in **(c)** corresponds to the HRTEM images of WSe2. **(f)** HAADF-STEM image of 1.93 wt% Ru–WSe2. **(g - i)** The EDS mapping of Ru, W, and Se, respectively.

(b)

Subfigure Token	Token Class	Subfigures Implied
(a) and (b)	parenthesis_02_and_alpha_02	[a , b]
(c)	parenthesis_02_none_alpha_01	[c]
(d, e)	parenthesis_02_comma_alpha_02	[d , e]
(c)	parenthesis_02_none_alpha_01	[c]
(f)	parenthesis_02_none_alpha_01	[f]
(g – i)	parenthesis_02_dash_alpha_03	[g , h , i]

(c) **Caption Text (Custom POS Tagging)**

```
(‘(a) and (b)’, ‘CAP’),(‘TEM images’, ‘NC’),(‘of’, ‘IN’),(‘1.93 wt\% Ru–WSe2’, ‘NC’), … ,(‘(d, e)’, ‘CAP’),
(‘The enlarged area’, ‘NC’),(‘denoted’, ‘IR’),(‘in’, ‘IN’),(‘(c)’, ‘CAP’),(‘corresponds’, ‘IR’),(‘to’, ‘IN’) …
```

(d)

Pattern #1	Interpretation	
(“CAP”, “!”, “NC”, “IN”, “*”, “.”)	“CAP”	- Start with a caption delimiter.
	“!”	- Do not record any text until …
	“NC”	- the next noun chunk that contains a …
	“IN”	- preposition immediately after.
	“*”	- Continue including all text until …
	“.”	- a full stop is detected.

(e) **Assigned Text From Pattern #1 (a and b), (c), …**

(a) TEM images of 1.93 wt% Ru–WSe2 | **(b)** TEM images of 1.93 wt% Ru–WSe2 | **(c)** HRTEM image of 1.93 wt% Ru–WSe2 …

Figure S2. **Demonstration of Custom Regex Methodology on Example Caption.** (a) The raw caption text. (b) Examples of *subfigure token* classifications. (c) Result of custom POS tagging. (d) Example sentence-level regex pattern and plain English interpretation. (e) Examples of the final assigned text for the selected subfigures.

Image Scaling Estimation

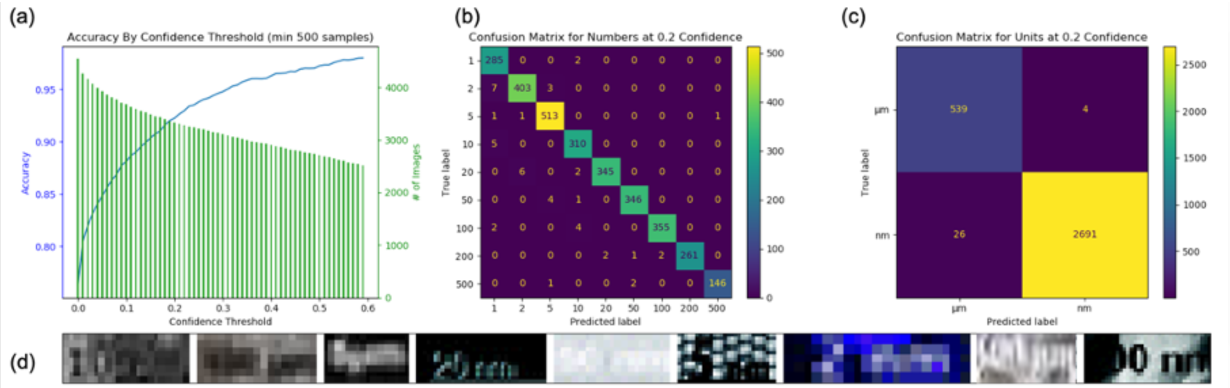

Figure S3. **Image Scaling Estimation Accuracy Examples.** (a) Estimation accuracy as a function of confidence threshold, including the number of images present at a given threshold. For all thresholds shown, there are at least 500 samples. When the confidence level associated with the scale bar label detection is ≈0.6, the overall accuracy (percentage of scale bar labels that are exactly right – both the number the unit) is > 0.95. (b, c) The confusion matrices highlight the accuracy of the predicted number and unit components for recognition of the scale bar label. Larger scales (*i.e.*, mm and cm) were not adequately represented in the training set, so they are not part of the test set. Both number and scale recognition accuracy is high at the 0.2 threshold (≈92% and ≈99% respectively for the labels shown). (d) Common instances of low-resolution and low-contrast conditions that are responsible for a majority of the prediction errors.

The image scaling estimation comprises two primary modules:

1. Scale Bar Detector (Faster RCNN with a Resnet-50-FPN backbone)

The *scale bar detector* was trained on the same dataset as the *figure separator*, with the objective of detecting scale bar lines and scale bar labels. A Gaussian blur with a 5×5 kernel size was randomly applied (with probability of .25) to training images. The Faster RCNN was pretrained on COCO train2017 and the backbone was pretrained on Imagenet. The Adam optimizer was used with a learning rate of 0.001, and a batch size of 32, and run for about 200 epochs. The non-maximum suppression method was used for inference. Figures were split between the test and train set by paper (all figures from a given paper will be in the same set to avoid leakage). The models were trained on a 4×NVIDIA Tesla V100 SXM2 with 32GB HBM2 and took about 4 minutes per epoch.

2. Scale Label Reader (Convolutional Recurrent Neural Network)

The CNN portion had 3 input channels (RGB) of 128×512 pixels, 22 output channels, and a sequence length of 32 and 6 layers with 64, 64, 64, 128, 128, and 128 output channels. This was fed into a bi-directional LSTM and then finally a fully connected output layer. The output was fed through LogSoftmax. CTC loss was used. Training proceeded for ≈1000 epochs with a batch size of 16. The model was trained and tested on a 4×NVIDIA Tesla V100 SXM2 with 32GB HBM2 with synthetic data. The synthetic data was generated by randomly placing random strings of 3-8 characters from the potential output channels on a set of textless microscopy images. Reported metrics were based on performance on the MTurk *figure separator* dataset, and took about 1 minute per epoch.

References

1. Mikolov, T., Sutskever, I., Chen, K., Corrado, G.S., and Dean, J. (2013). Distributed representations of words and phrases and their compositionality. Advances in neural information processing systems 26. 10.48550/arXiv.1310.4546.

2. Blei, D.M., Ng, A.Y., and Jordan, M.I. (2003). Latent dirichlet allocation. Journal of machine Learning research 3, 993-1022.

3. Mimno, D., Wallach, H., Talley, E., Leenders, M., and McCallum, A. (2011). Optimizing semantic coherence in topic models. pp. 262-272.
