# Supplementary material for: EXSCLAIM!: Harnessing materials science literature for self-labeled microscopy datasets
Source: Patterns (N Y). 2023 Sep 30;4(11):100843. doi: 10.1016/j.patter.2023.100843 (PMC10682750; doi:10.1016/j.patter.2023.100843)
Supplement: Document S2. Article plus supplemental information [file mmc2.pdf]

Article

Patterns

EXSCLAIM!: Harnessing materials science literature for self-labeled microscopy datasets

Highlights

- Software pipeline for creating self-annotated imaging datasets from science journals
- Design uses configurable modules to scrape and process scientific figures/captions
- Initial applications focus on materials microscopy, but techniques apply generally
- Validation provided for figure separation and NLP caption distribution accuracy

Authors

Eric Schwenker, Weixin Jiang, Trevor Spreadbury, Nicola Ferrier, Oliver Cossairt, Maria K.Y. Chan

Correspondence

mchan@anl.gov (M.K.Y.C.), e.schwenker89@gmail.com (E.S.)

In brief

This study presents a software pipeline that uses computer vision and natural language processing approaches for automatic extraction, separation, and caption-based natural language annotation of images (EXSCLAIM!) from scientific literature and demonstrates its effectiveness in constructing a self-labeled electron microscopy dataset of nanostructure images. EXSCLAIM! is developed around materials microscopy images, but the approach is applicable to other scientific domains that produce high volumes of publications with joint image-text data (e.g., graphs, schematic illustrations, etc.).

Schwenker et al., 2023, Patterns *4*, 100843
November 10, 2023 © 2023 Argonne National Laboratory and The Author(s).
Published by Elsevier Inc.
https://doi.org/10.1016/j.patter.2023.100843

CellPress

Patterns

CellPress
OPEN ACCESS

Article

EXSCLAIM!: Harnessing materials science literature for self-labeled microscopy datasets

Eric Schwenker,[1,2,*] Weixin Jiang,[1,3] Trevor Spreadbury,[1,3] Nicola Ferrier,[4] Oliver Cossairt,[3] and Maria K.Y. Chan[1,5,*]
[1]Center for Nanoscale Materials, Argonne National Laboratory, Argonne, IL 60439, USA
[2]Department of Materials Science and Engineering, Northwestern University, Evanston, IL 60208, USA
[3]Department of Computer Science, Northwestern University, Evanston, IL 60208, USA
[4]Mathematics and Computer Science, Argonne National Laboratory, Argonne, IL 60439, USA
[5]Lead contact
*Correspondence: mchan@anl.gov (M.K.Y.C.), e.schwenker89@gmail.com (E.S.)
https://doi.org/10.1016/j.patter.2023.100843

THE BIGGER PICTURE Due to recent improvements in image resolution and acquisition speed, materials microscopy is experiencing an explosion of published imaging data. The standard publication format, while sufficient for data ingestion scenarios where a selection of images can be critically examined and curated manually, is not conducive to large-scale data aggregation or analysis, hindering data sharing and reuse. Most images in publications are part of a larger figure, with their explicit context buried in the main body or caption text; so even if aggregated, collections of images with weak or no digitized contextual labels have limited value. The tool developed in this work establishes a scalable pipeline for meaningful image-/language-based information curation from scientific literature.

1 2 3 4 5 **Proof-of-Concept:** Data science output has been formulated, implemented, and tested for one domain/problem

SUMMARY

This work introduces the EXSCLAIM! toolkit for the automatic extraction, separation, and caption-based natural language annotation of images from scientific literature. EXSCLAIM! is used to show how rule-based natural language processing and image recognition can be leveraged to construct an electron microscopy dataset containing thousands of keyword-annotated nanostructure images. Moreover, it is demonstrated how a combination of statistical topic modeling and semantic word similarity comparisons can be used to increase the number and variety of keyword annotations on top of the standard annotations from EXSCLAIM! With large-scale imaging datasets constructed from scientific literature, users are well positioned to train neural networks for classification and recognition tasks specific to microscopy—tasks often otherwise inhibited by a lack of sufficient annotated training data.

INTRODUCTION

Journal articles have long been the standard for communicating important advances in scientific understanding. As sophisticated measurement and visualization tools render scientific communication more intricate and diverse, the visual presentation of scientific results in these articles is noticeably more complex.[1] With this complexity, which is often a byproduct of the "compound" layout of the figures (i.e., figures containing multiple embedded images, graphs, illustrations, etc.), the meaning of each standalone image is not always apparent. The result is that individual images are not only unsearchable but that the effort required to extract them into a machine-readable format is significant. This plays a major factor in the relative scarcity of general materials characterization images in the development and testing of new algorithms in deep learning (DL), the use of deep neural networks to learn representations of input data (often images) that reveal important characteristics of its content or overall appearance. The current surge of interest in DL stems from recent success in applications such as facial recognition,[2] self-driving cars,[3] and complex game playing.[4] Much of this success is the byproduct of having large labeled datasets available for training,[5] and in order for current materials imaging classification and recognition tasks[6–13] to reap the benefits afforded by DL pipelines, having access to substantial labeled data is crucial. Fortunately, the incentive to publish is nearly ubiquitous across

© 2023 Argonne National Laboratory and The Author(s). Published by Elsevier Inc.
This is an open access article under the CC BY license (http://creativecommons.org/licenses/by/4.0/).

all scientific disciplines, and with a mechanism for automatic dataset construction that includes both separating out individual images from compound figures as well as providing concise annotations describing key aspects or classification of the image content, much more of the scientific imaging data in literature could be utilized for training and developing DL models.

The effort to automate the construction and labeling of datasets from general web data has garnered broad attention from the computer vision, language technologies, and even chemistry/materials informatics communities.[14–17] In chemistry and materials informatics, text-mining tools adapted for "chemistry-aware" natural language processing (NLP) have been developed to create datasets of material properties and synthesis parameters from journal article text.[18–22] In computer vision, labeled image data have been directly obtained from image search engines, and additional pipelines have been constructed to ensure scalability, accuracy, and diversity in the image collections.[17] For scientific literature data specifically, tools using a combination of topics learned from abstracts in conjunction with custom filtering on chemical information have been shown to facilitate the systematic collection of figures pertaining to a particular line of scientific questioning (e.g., what images pertain to common microstructural characterizations for glasses?[23]). Unfortunately, these approaches can be problematic for scientific figures, which are often organized as collections of images displayed in a compound layout. Hence, tools that use figures without extracting the images comprising them tend to lose their specificity.

Current works that do address this layout problem (what we refer to as problem of "figure separation") rely on hand-crafted rule-based approaches[24–27] or adapt neural networks to interpret figure separation as an object detection problem.[28,29] While hand-crafted techniques are generally effective for sharp image boundaries, and neural networks capture irregular image arrangements, neither approach considers explicit references to the caption text in context of the separation. Despite efforts to advance figure separation and summarization related to automatic dataset creation,[24,25,30–33] tools that do this in query-to-dataset fashion, capable of extracting individual images from figures, classifying image content, recognizing scaling information, and assigning a relevant descriptive label based on specific user search requirements, are currently lacking.

In this work, we develop a software pipeline for the automatic extraction, separation, and caption-based natural language annotation of images (EXSCLAIM!) from scientific figures and demonstrate its effectiveness in constructing a self-labeled electron microscopy dataset of nanostructure images. While the initial application focus of EXSCLAIM! is materials microscopy images, the methods comprising the pipeline are general and thus applicable to other scientific publication domains producing high volumes of image data.

RESULTS

Design overview

The goal of EXSCLAIM! is to (1) enable comprehensive keyword searches for scientific figures within open-source journal articles and (2) facilitate the extraction and pairing of images from within figures to the appropriate descriptive keywords and phrases from the caption text. Figure 1 is an overview of the software pipeline, highlighting the role that the journal scraper, caption distributor, figure separator, and dataset constructor modules serve in transforming search queries into structured self-labeled image datasets.

Search query

The search query defines the scope and extent of the search and is a JSON object with attributes populated by the user. The journal_family, sortby, and save_format attributes establish the publishing source, the ranking method used to retrieve content, and the desired save formats for pipeline outputs, respectively. The keyword attributes and synonym lists provide the main topics and ways to expand them with relevant variations (i.e., words from the synonym lists that match caption text are mapped to their associated keyword in the caption distributor module).

Journal scraper

The journal scraper extracts relevant article URLs from a collection of individual searches using all pairwise combinations of the keyword attributes and associated synonym list entries in the search query. With an ordered list of open-source article URLs, the scraper uses the Python Requests library[34] to handle the HTTP requests, returning article content with caption data and an associated URL for each figure. Currently, the journal scraper supports parsing for *Nature*, *American Chemical Society* (ACS), and *Royal Society of Chemistry* (RSC) journals but can easily be extended to additional publishing sources. More details are available in the supplemental notes. On average, extracting all figures and captions, as well as parsed versions of the main-body text, takes approximately 12 s per article on a single-core processor. Additionally, although open-source articles are explicitly targeted in the design and testing of the current tool, if a user has the credentials to access subscription-based content, they also have the capacity to extract such content with EXSCLAIM! Note that the scraping is targeted and driven by a keyword search (i.e., not indiscriminate) and therefore adheres to journal access rules.

Caption distributor

With figure-caption pair information recorded, the next step is to distribute subsequences of caption text to the subfigures they reference. This is achieved using a combination of (1) customized part-of-speech (POS) tagging, configured to deconstruct caption text into small units (tokens) based on POS or structural function (e.g., subfigure label, cross-reference, etc.), with (2) regular expression-style pattern matching, tuned to extract sentence-level phrases for typical subfigure image descriptions from sequences of custom POS tokens. All POS tagging and token customization is performed using the spaCy library for NLP.[35] Additional implementation details for these custom regex methods are provided in the experimental procedures.

The result of proper caption distribution is a phrase such as "Au nanoparticles deposited on (a) n- and (b) p-type Si(100) substrates" being distributed to subfigure (a) as "Au nanoparticles deposited on n-type Si(100) substrates" and to (b) as "Au nanoparticles deposited on p-type Si(100) substrates." On average, caption distribution takes approximately 0.65 s per caption on a single-core processor. While transformer-based language models exist for closely related domains,[36–38] there is an absence of available datasets to perform the fine-tuning necessary to achieve

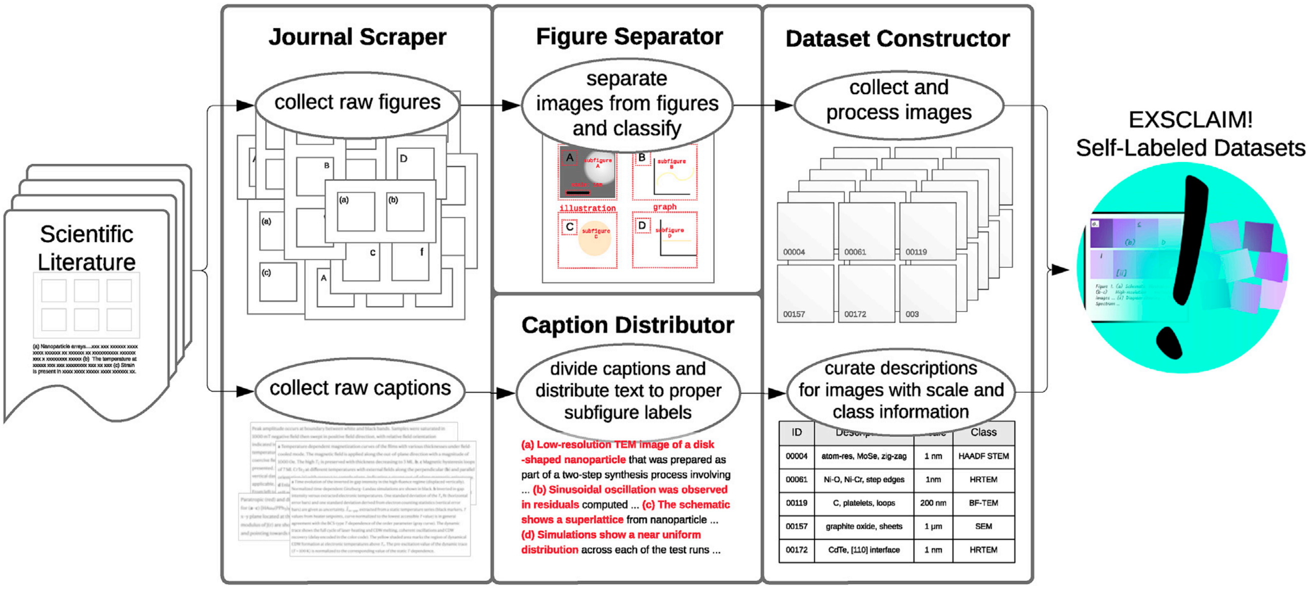

Figure 1. Overview of the EXSCLAIM! pipeline

The journal scraper extracts raw figure-caption pairs from journal articles. The caption distributor divides caption text into segments that are consistent with the images in the figure, reducing them to single keywords if possible (i.e., self-labeling). The figure separator computes bounding boxes that separate and classify the individual images from the full figure. And finally, the dataset constructor processes all prior information to create a structured self-labeled dataset.

caption distribution at a scale. Additionally, with the rule-based approach, a user can more easily modify phrase extraction to better control precision for the target research domain.

Finally, in addition to the keyword attribute/synonym list pairs provided as part of the search query, the caption distributor includes a built-in dictionary of common materials science characterization keywords with synonyms.[39] Any of these dictionary or search query terms appearing in the segmented caption text explicitly are mapped to their appropriate keyword topic and included as a class label for the image they are paired with in the next steps. This built-in dictionary can easily be extended as the user sees fit.

Figure separator

A scientific figure is often composed of multiple individual images that must be separated before further processing and pairing with caption text. This separation step involves subdivision of the extracted figures into "master images" according to what we establish as the master-dependent-inset (MDI) modeling paradigm. In the MDI paradigm, the master image is defined relative to a subfigure label (e.g., (a), (b), etc.), and the subfigure label is the functional element bridging the visual image content to the text describing it. All visual components belonging to the complete entity referenced by the subfigure label are collectively referred to as the "master image." Figure 2 provides a detailed view of the MDI model applied to a standard figure. The subfigure (b) in Figure 2 highlights the need for a "master image" classification, as it is referencing more than one distinct image. Master images containing a single visual component are further classified as "microscopy," "graph," "diffraction," or "illustration," and those with multiple visual components are considered "parent" images. This style of figure separation is the primary task of the figure separator, which follows a two-stage framework outlined in Jiang[40]; it takes an average of 4.5 s to completely separate the images from a figure on a single-core processor. Further, the authors also developed a tool Plot2Spectra for the automated digitization of "graph" style subfigures, as reported by Jiang et al.[41]

The first stage of figure separation identifies subfigure labels within the compound figure. This is achieved using a combination of object localization (YOLOv3-style object detector[43]) and object recognition (ResNet-152[44]) neural networks. In the second stage, binary masks are created as visual anchors for subfigure locations in the full figure. The binary mask is then concatenated with the standard RGB input channels and fed into the master image detection module. Taken together, these networks locate and classify master images within the figure, preserving their association to the appropriate subfigure label. The focus of this work is primarily on the recognition of "master images," though a task such as "inset recognition" naturally follows. Further model details are available in the supplemental notes.

Dataset constructor

The dataset constructor serves as the final information consolidation step, using the extracted information from the preceding steps to download the individual image locally and to pair with a CSV file or local database server. The CSV file or database contains row entry descriptions (i.e., the subsequences of separated caption text) and keywords for each image. Additionally, in this final pipeline step, images annotated with scaling information are further processed to define scale explicitly. This estimation of image scaling is accomplished using another series of custom neural networks (method and model construction details are provided in the experimental procedures). With suitable estimation of object magnification, users can begin quantifying image content based on size, and searches through resulting databases can be refined to a particular size range.

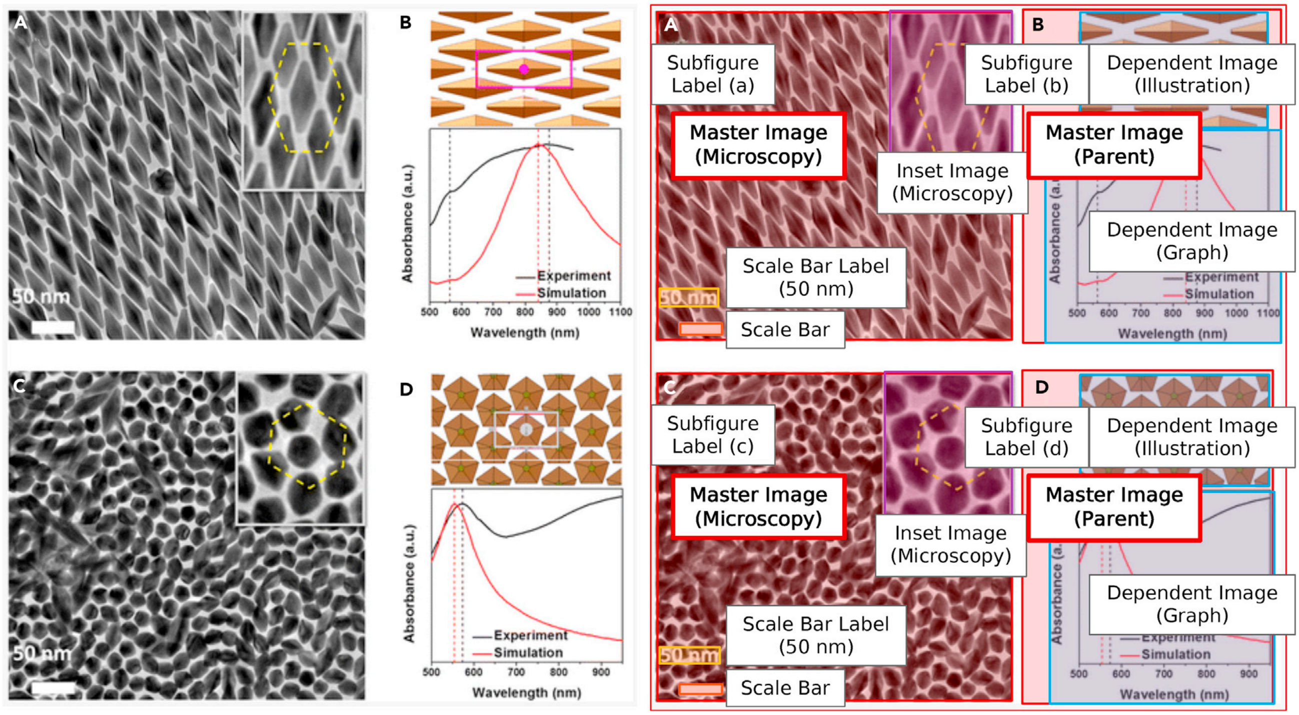

Figure 2. Master-dependent-inset (MDI) Model
The left panel is the initial figure before annotation, and the right is a copy that has been properly annotated according to the MDI model. The presence of a subfigure label is necessary for an image to be given a "master image" designation. Because the master image for subfigure b governs two distinct images ("illustration" and "graph"), it is classified as a parent. Additional image features such as the scale bar and scale bar label are also identified. Adapted with permission from Shi and co-authors.[42] Copyright 2016 American Chemical Society.

Pipeline module evaluations

Several components of the EXSCLAIM! pipeline must be considered when evaluating overall performance. Here, we (1) measure figure separator classification performance using precision and recall metrics obtained on a reference dataset, (2) examine various caption distribution scenarios, quantifying accuracy for instances where a single keyword is used to describe the image, and finally (3) provide suggestions for how to create new labels or general topics to associate with images that are left un- or under-annotated.

Figure separator validation

Classification accuracy for the figure separator is validated using precision and recall. The validation dataset[45] includes images annotated according to the MDI paradigm and is available from the Materials Data Facility.[46,47] For this analysis, positive predictive value (precision) is prioritized over a measure of completeness (recall). This assumes that the opportunity cost of failing to include relevant images is low and does not affect the integrity of the dataset as much as images that are incorrectly classified. The confusion matrices in Figure 3 provide both a clear visualization of precision and recall, as well as context for what types of errors are being made. They are given in both a "no threshold" (Figure 3A) and a "high threshold" (Figure 3B) condition. With "no threshold," the most likely classification, regardless of magnitude, is accepted. With "high threshold," only classification values greater than or equal to 0.99 are accepted. The "microscopy," "graph," and "diffraction" predictions are all favorable from a precision perspective, as all values exceed 0.80 and get as high as 0.98 in the "high threshold" case for "graph."

The dataset is inherently imbalanced (i.e., "microscopy" is included explicitly in the search query, "graph" visualizations are more prevalent than the other classes in scientific articles, etc.). As such, we also provide a measure of classification quality in terms of the Matthews correlation coefficient.[51] The Matthews correlation coefficient (MCC) is a standard classifier performance metric in machine learning and statistics and is often regarded as a more informative response when compared to accuracy for classes of very different sizes (which is susceptible to overoptimistic estimation of the classifier's ability on the majority classes). It is more informative in the sense that good results must be obtained on all categories of prediction in the confusion matrix (i.e., true positives, true negatives, false positives, false negatives) to yield a high positive score (+1: perfect classification, 0: inefficient classification, −1: perfect misclassification). Because the "high threshold" condition excludes many results by design, we only examine the MCC for the "no threshold" result. MCC scores for "microscopy" and "graph" are 0.73 and 0.84, respectively. Overall, precision scores for the "microscopy" and "graph" classes are high, and the MCC values are well above "inefficient." Additional details on figure separator evaluation as well as training/validation dataset construction can be found in the experimental procedures.

Finally, Figure 3C highlights some interesting false positive examples where "diffraction," "illustration," and "parent" classes are incorrectly classified as "microscopy." In the example on the left, the diffraction patterns show periodicity resembling

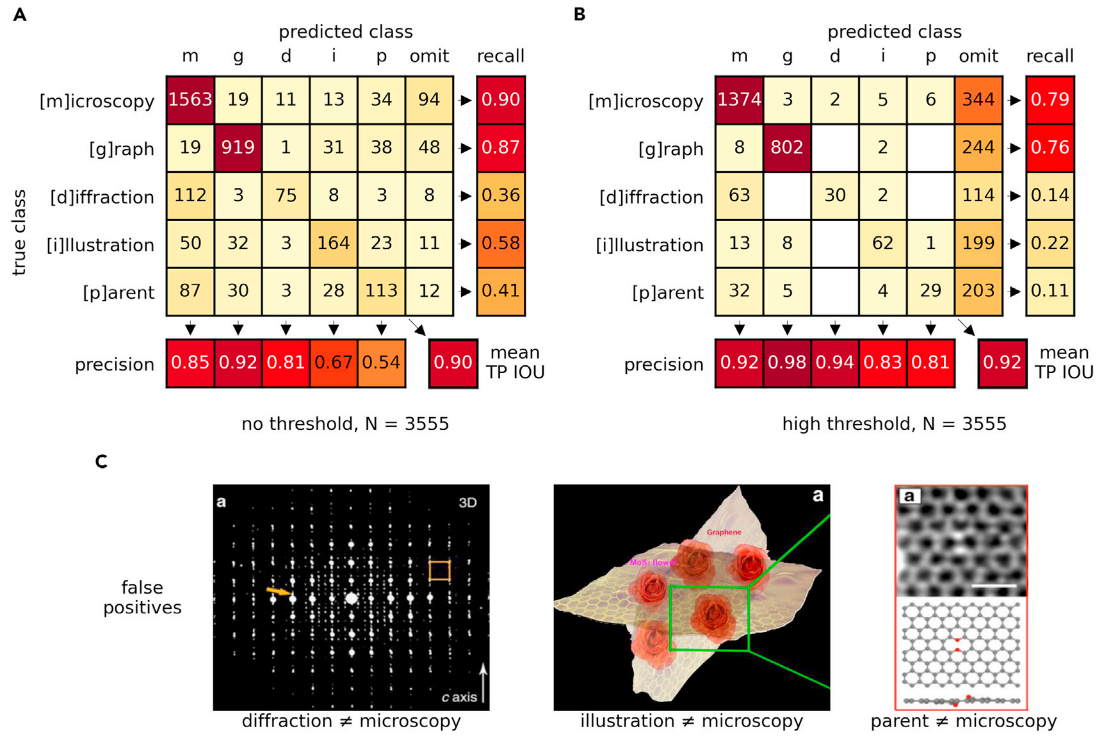

Figure 3. Figure separator classification accuracy

(A and B) The confusion matrices highlight the nature of the mistakes made in each classification scenario at two confidence thresholds, (A) no threshold and (B) high threshold, for n = 3,555 images. In both cases, the precision scores are adequate, particularly in the case of the "microscopy," "graph," and "diffraction" images. Recall suffers across the board as correctness is emphasized over completeness in the design of the pipeline. The "omit" class is included for sake of completeness and represents classes such as "photograph" and "unclear" that exist in the dataset but were not part of the training for the figure separator.
(C) Images highlight some of the more easily rationalized examples of false positive "microscopy" classifications. Citations for images in (C), from left to right, are Willhammar et al.,[48] Zhao et al.,[49] and Hofer et al.[50]

atomic-resolution microscopy images, so the "microscopy" class assignment is understandable. In the middle, the illustration maintains some features commonly associated with a microscopy image (i.e., a darker background, grainy texture, etc.), but when the content of the image is considered as a whole, "microscopy" is noticeably incorrect. Lastly, the far-right example (Figure 3C) does contain a microscopy image, but because the image below it does not contain a subfigure label and is "semantically" tied to the microscopy image in (a), the most appropriate classification for this image is "parent."

Electron microscopy images of nanostructures

To evaluate caption distributor performance, we construct an example use case that involves creating an annotated dataset of electron microscopy images of nanostructures and analyze the accuracy of the specific keywords assigned to the extracted images. This example dataset is constructed from open-source *Nature* articles using a "sort by relevance" search. The search query is formed from the following collections of keyword attributes (synonym lists are omitted for brevity): ("electron microscope," "electron microscopy") and ("nanoparticle," "nanosheet," "nanoflake," "nanorod," "nanotube," "nanocrystal," "nanowire," "nanosphere," "nanocapsule," "nanofiber"). This query mimics a wildcard-style search for nanostructures imaged in an electron microscopy modality. In this example, EXSCLAIM! returned a total of 13,450 open-source articles with 83,504 figure-caption pairs. To better facilitate manual validation of retrieval performance on master images classified as "microscopy," quantification is limited to articles in the top 10% of the relevancy ranked list, which amounts to a total of 28,962 individual images. The full dataset[52] is available from the Materials Data Facility.[46,47]

Unsurprisingly, the 28,962 validation images contain a substantial number of assigned "microscopy" and "graph" classes. Figure 4A highlights the distribution of predicted image classes in the top 10% of retrieved articles and uses the color-coded divisions of each bar to depict a level of classification confidence for each prediction (i.e., exceeding the "high threshold" amounts to a "highest" confidence prediction). The abundance of graphs and microscopy images, taken with the precision metrics from Figure 3, implies that a large fraction of the high-confidence classifications, particularly in the case of microscopy images and graphs, are likely to be actual instances of "microscopy" and "graph" classes (e.g., precision scores of 0.92 and 0.98, respectively, for the "high-threshold" condition). Having a sizable number of confident predictions is essential in the effort to create a reliable dataset at scale.

Next, we analyze the proportion of images receiving proper processed text strings and further examine the frequency of keyword extractions from these strings. Figure 4B identifies four categories of label types that distinguish these scenarios. In particular, the "label uas" (label unassigned) category represents separated images

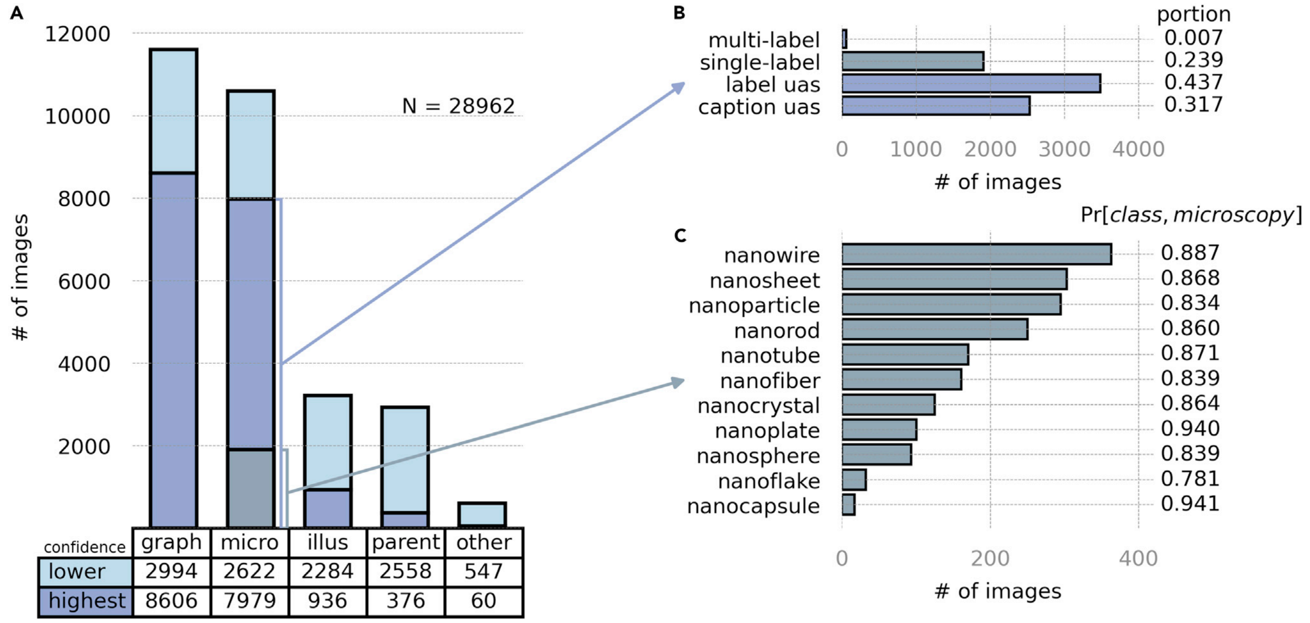

Figure 4. Distribution of search results for nanostructures query

The example query is used to extract electron microscopy images of general nanostructures from *Nature* journals. The bar plot in (A) shows the distribution of image types extracted at two different thresholds. The bar plots in (B) and (C) further subdivide the population of high-confidence "microscopy" images. In (B), the distribution of label types is recorded. In this context, "single-label" and "multi-label" refer to the existence of a keyword label. "Label uas" (label unassigned) means that caption text has been distributed to the image but that no keyword label from the initial query exists. "Caption uas" (caption unassigned) refers to a scenario where the caption distributor was not able to confidently distribute a proper substring to caption text. The bar plot in (C) represents the distribution of labels in the top 10% of retrieved "microscopy" images and provides an estimate of the joint probability that an image classified as "microscopy" and given one of the "class" names listed along the vertical plot axis is a true microscopy image represented by the given "class" label.

that have received a proper string of the caption text as a description but do not contain any of the keyword attributes from the search query. If this is problematic from a throughput perspective, it is advised that more search terms should be included as part of the initial search query or the built-in dictionary. Note that to provide the most representative data possible for how a user-defined search query drives keyword extraction, words from the built-in dictionary were omitted as possible keywords in the distributed caption texts. The "caption uas" (caption unassigned) category contains separated images that have not received any portion of the caption text. Captions that are left unassigned typically have sentence structure complexities, such as multiple compound subjects, long intervening phrases, etc., that make rule-based caption distribution ambiguous. And while the percentage of "caption uas" images is high, this is expected, as the data abundance assumption and "no information is better than bad information" design principles underscore many of EXSCLAIM!'s design choices. The final conditions, identified in Figure 4B, are the "single-label" and "multi-label" conditions, which represent images with assigned caption text, containing explicit reference keyword attributes from the search query. The quality of this processed text is important because it gives the extracted images their specificity.

Finally, the "single-label" category is further dissected in Figure 4C, and its relation to the initial full set of extracted "microscopy" images is emphasized with the gray coloring in Figure 4A (1,909 of the images in the high-threshold group, or ~24%, are "single-label"). The bar chart itself shows the frequency of single nanostructure keyword attributes from the initial search query (i.e., "nanoparticle," "nanosheet," "nanoflake," etc.) present in the text strings distributed to each subfigure image. All 1,909 "single-label" "microscopy" image-keyword pairs were manually verified against the full caption text to create a ground-truth dataset of image-keyword pairs. The values recorded in the right margin of the bar plot in Figure 4C represent the joint probability that a correct prediction of "microscopy" alongside a correct keyword classification will occur simultaneously for a given "single-label" image. This provides a general measure of confidence among all image-keyword pairs, as higher joint probabilities indicate higher likelihoods that both conditions are true. The average joint probability across all classes is ~87%, which means that of the 1,909 single-label "microscopy" images, ~1,660 are true microscopy images with correct keyword labels. Extrapolating beyond the top 10% of retrieved articles, there are ~4,300 "microscopy" images total with a single keyword attribute explicitly related to the initial search query; thus, it is reasonable to estimate a yield of >3,700 high-confidence "microscopy" images with the appropriate keyword label—all from a single journal family.

Current caption distribution prioritizes identification of appropriate keywords over language correctness, so only keyword accuracy is factored into the ground-truth comparisons in Figure 4. However, there are several examples where the distributed caption is grammatically sufficient, and this even extends to scenarios where the caption text contains non-contiguous segments. For example, in Figure 5A, the distributed caption uses all the text from the subfigure identifier (b) to the period signifying the end of the sentence; however, Figures 5B and 5C show that the current

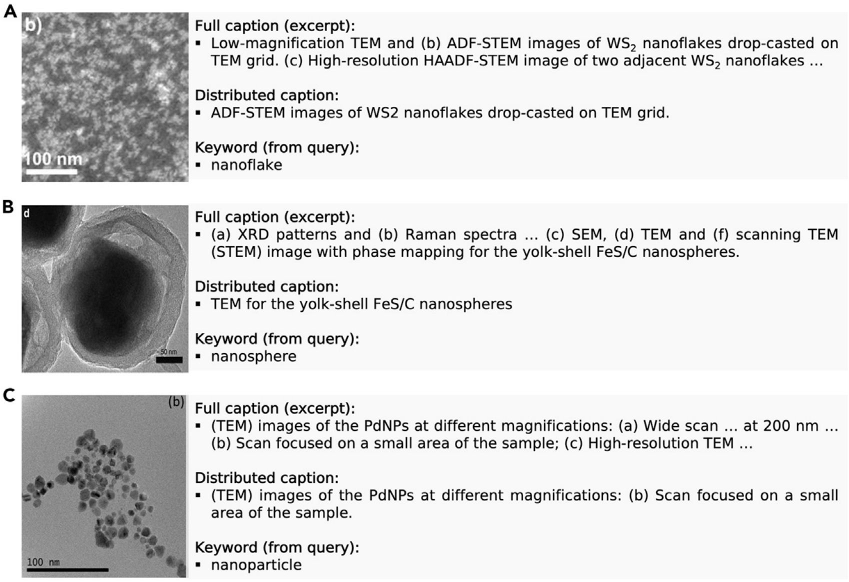

Figure 5. Example image/caption pairings

The extracted images contain both a keyword attribute shared with the initial search query, as well as a grammatically sufficient sequence of distributed caption text. Some caption distribution examples are simple, as in (A), where all the distributed words are linearly connected. However, the current caption distributor is also designed to capture more complex structural dependence relationships (B and C) where the subject is separated from the text completing the full consistent description. The citations for image-caption text excerpts are (A) Mastria et al.,[53] (B) Wang et al.,[54] and (C) Tripathi and Chung.[55]

method can identify the structurally closest text sequence, even when it is not spatially the closest. For example, in Figure 5C, the subject of the sentence "TEM images" is not matched to the spatially closest descriptions (adjectives) or even the closest preposition but rather to the preposition at the end of the sentence. Structurally, this makes sense and is the proper division of this text.

Annotation expansion with NLP

We have demonstrated the effectiveness of the EXSCLAIM! tool in situations where keywords are extracted from the caption text, and we even show that in some situations when the structure of the caption is complex, the caption distributor is still capable of extracting sentences and phrases in a way that preserves the intended meaning. However, with the current design, any topics that are prominent in the returned set but not explicitly specified in the search query or built-in dictionary are ignored. The advantage of treating the problem of dataset construction in this manner is that it ensures a high degree of relevance in the images that are assigned a keyword label, but it does limit the scope of the self-labeling task in general. To this end, we first suggest including more search terms in the query (synonym list attributes in particular) to better capture the breadth of language used to describe the concepts that the user deems important. Here, though, we also explore how NLP tools can be leveraged to transform the phrases or sentences of the distributed caption text into a series of relevant, hierarchical labels for each image they are referencing. This gives users another option of generalizing this labeling to other scientific topics that are not explicitly part of the initial search query.

Figure 6 provides examples of how an image-caption pair is transformed into a series of hierarchical labels that both describe and contextualize the image content. In the examples, "caption" labels are determined using an iterative word dropout approach that removes words that are semantically furthest away from the center of the current group of caption words. The "abstract" labels are the 2–3 words closest to the center of the combination of abstract and caption words and typically provide context for the image outside of what is explicitly defined in the caption. Finally, a statistical model that uses abstracts and introduction texts to assign the best "topic" label to the document containing the image provides further context-aware labeling for the image. Figure 6 shows several compelling examples of how this approach can provide useful contextual labels for the images from language outside those found explicitly in the distributed caption. For example, we learn things that we can confirm visually, such as the fact that nanorods are elongated and cylindrical (Figure 6A), as well as things an expert might know that reveal the function of the image content, such as Fe/FeO nanocrystals function as nanotherapeutics (Figure 6B); RuIrZnOx-U nanoboxes are synthetic as opposed to biological catalysts (Figure 6C); austenite grains describe the "microstructure" of the image (Figure 6D); and nanocrystals are MA lead halide perovskites (numerical characters get stripped in the text preprocessing, so "mapbbr" refers to MAPbBr3) (Figure 6F); even in Figure 6E, where the abstract context is more redundant than unique or complementary, the topic provides useful context for where the image content appears from an application perspective. Overall, the statistical topic modeling approach with semantic word similarity comparisons provides a solid backbone for the self-labeling imaging effort. Further model and performance details can be found in the supplemental notes.

DISCUSSION

Currently, the rule-based caption distributor drives the assignment of proper substrings of text to the extracted subfigure images, focusing primarily on high-precision distribution, and the NLP approaches provide an additional source of image labels from context external to the caption text. While rule-based caption distribution provides a straightforward way to model and update specific syntax complexities, it is difficult to generalize across all published caption text because there are no universal standards regarding caption syntax. This is largely the reason why despite its measured success in situations where substrings are distributed and keywords extracted (as reported with the high average joint probability scores), there is still nearly a third of the extracted images not receiving a substring of text from the caption distributor. This is where transformer-based NLP models[63–65] stand to

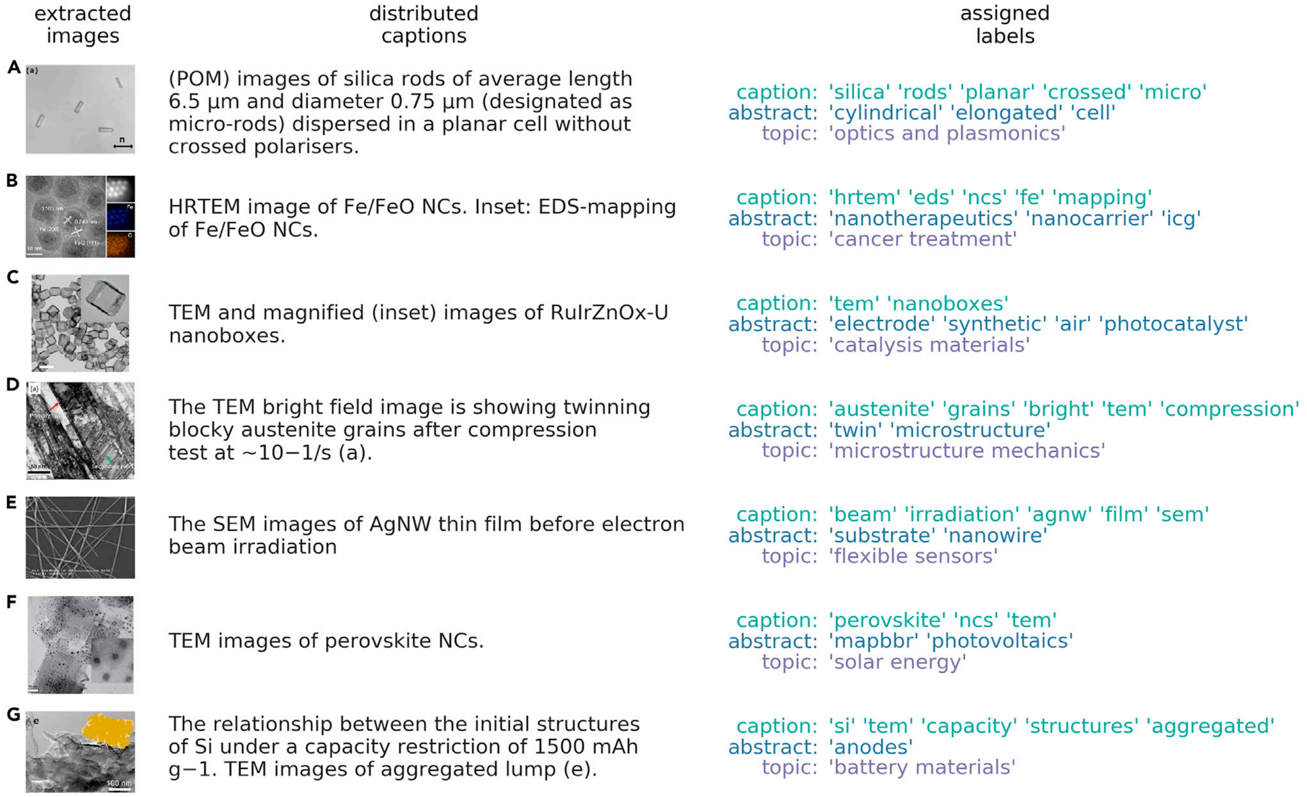

Figure 6. Examples of hierarchical label assignment from NLP

Examples of hierarchical label assignment for images containing a properly distributed caption. For each image, the caption labels are limited to caption text only. Conversely, the abstract labels are free to draw additional relevant words from the abstract text, and the topic labels come from the human-assigned topic names from the statistical topic summaries. Citations for image-caption text are (A) Pujala and Dhara,[56] (B) Wang et al.,[57] (C) Zhuang et al.,[58] (D) Hossain et al.,[59] (E) Hong et al.,[60] (F) Huang et al.,[61] and (G) Kasukabe et al.[62]

make the greatest contribution to the overall pipeline, as they have proven effective in accurately generalizing over a large range of contexts in many of the NLP subtasks comprising an improved, more generalized caption distributor model (i.e., named entity recognition, text summarization, *etc.*). Fortunately, exceptional performance in these tasks can often be achieved with domain-specific fine-tuning of pre-trained models, and the existing caption distributor can be used to seed some of the initial annotation required in the construction of new domain specific datasets (i.e., make use of the existing high-precision annotations). Moreover, the authors recognize the great potential of the existing pipeline to facilitate multi-modal learning by using the figure separator and the caption distributor in a semi-supervised feedback loop to align language and image representations in the same embedding space. Both extensions to incorporate transformer-based NLP models and multi-model embeddings are under development.

Conclusion

We present EXSCLAIM!, a software pipeline for the automatic extraction, separation, and caption-based natural language annotation of images from scientific figures. In this work, we detail the specific extraction tools and provide quantitative performance measures for image classification and keyword labeling accuracy on both a crowdsourced-labeled dataset and an extracted dataset of nanostructure figures from *Nature*-family journals. In addition, we provide discussions and useful model implementations aimed at assigning additional image labels from complete sentence text. Successful consolidation and self-labeling of images from scientific literature sources will not only enhance the navigation and searchability of images spanning materials, medical, and biological domains but is a vital first step toward introducing scientific imaging to the canon of training datasets for state-of-the-art algorithms in computer vision and NLP.

EXPERIMENTAL PROCEDURES

Resource availability

Lead contact

The lead contact is Maria K.Y. Chan (mchan@anl.gov).

Materials availability

The work did not generate any physical materials.

Data and code availability

The validation dataset for figure separator classification accuracy in Figure 3 is available via the Materials Data Facility (Materials Data Facility: https://doi.org/10.18126/a6jr-yfoq). The annotated dataset of nanostructure microscopy images, used to analyze the accuracy of keywords assigned to the extracted images in Figure 4, is similarly available (Materials Data Facility: https://doi.org/10.18126/v7bl-lj1n). EXSCLAIM! can be found on GitHub (https://github.com/MaterialEyes/exsclaim) and on PyPI (https://pypi.org/project/exsclaim). A static version of the code is also available on Zenodo (https://zenodo.org/record/8260540, https://doi.org/10.5281/zenodo.8260539).

Custom regex matching

Caption distribution relies heavily on custom regular expression-style pattern matching. Regular expressions (regexs) are sequences of characters that describe patterns of text and are used at the beginning of caption distribution to identify instances where subfigures are referenced in the caption. These instances are referred to as subfigure tokens, and the pattern of opening punctuation, followed by a collection of internal alphanumeric characters, proceeded by some closing of the opening punctuation, is one of several pattern options considered when defining subfigure tokens.

Consider the example provided in Figure S2. The bolded subfigure tokens are recognized within the raw caption (Figure S2A) and are classified to determine whether further enumeration is needed to represent all the subfigures implied (Figure S2B). Namely, "(a) and (b)" is classified as parenthesis_02_and_alpha_02, where parenthesis identifies the opening punctuation, 02 signifies punctuation both before and after each internal character, "and" is the internal delimiter between characters inside the punctuation, alpha is the internal character type, and the final 02 denotes the number of explicit characters in the token. This token, with and as the internal delimiter, references two subfigures, whereas a token such as "(g - i)" with a dash as the internal character delimiter implies reference to all subfigures in the interval range (i.e., g, h, and i).

From here, the subfigure tokens become the anchor points around which caption text is distributed. To distribute the caption text, the sentence is again tokenized but this time by assigning the proper POS to each word (Figure S2C). This is a common preprocessing step in NLP pipelines referred to as POS tagging. For this custom implementation of POS tagging, subfigure tokens (CAP) are identified alongside standard POS tags, such as singular noun (NN), verb (VB), preposition (IN), etc. In addition to the custom tagging of subfigure tokens, noun phrases are further consolidated into noun chunks (NC), and a tag for internal referencing is added (IR), which marks an instance where a subfigure token is mentioned within the scope of another. With the caption now a sequence of customized POS tokens, each sequence is matched against a dictionary of common sentence patterns to find all text associated with the discussion of a given subfigure. In addition to looking for sequences of POS tokens, these patterns utilize "include all '*'" and "exclude until '!'" tokens, and a maximum count on the number of subfigure tokens in each sentence, to achieve both flexibility and specificity when assigning text to a subfigure. An example pattern is included alongside its plain English interpretation in Figure S2D. The text surrounding the "(a) and (b)" token fits this pattern and, as a result, is distributed accordingly (Figure S2E).

Image scaling estimation

The method used to estimate image scaling relies on a two-stage neural network. First an object detection neural network (Faster R-CNN[66]) is used to detect the bounding boxes of scale bar labels and scale bar lines that exist in a given figure. Next, the detected scale bar labels are fed into a convolutional recurrent neural network (CRNN[67]) in order to perform text recognition, which makes the scale bar label text machine readable. Further model details are available in the supplemental notes. The result of the CRNN is processed by a rule-based search to ensure the output is a valid scale bar label (i.e., a number followed by a unit). Multiple scale bar lines and scale bar labels in a single figure are matched by assigning the scale bar labels to scale bar lines greedily based on the distance between the center of their respective bounding boxes. Each matched scale bar-scale bar label pair is assigned to the subfigure in which it is contained. Using the length of the scale bar line, in pixels, and the subfigure and the scale bar label text, the real space size of the subfigure can be determined. Some accuracy measures for image scaling estimation are included in Figure S3.

Figure separator training/validation dataset

This section details the construction of the dataset[45] used to train and validate the figure separator. Because the demands of the MDI task are unique and figure separation does not fall explicitly within the canon of standard computer vision tasks, we needed an approach to create a dataset that we could use for training and validation of the figure separator. For this, we used the crowdsourcing platform from Amazon called Mechanical Turk (MTurk). Though proper interpretation of a scientific image often requires an expert to understand the nuances of the image content, identifying where the master images are located, as well as their proper classification, can be formulated so that those without a rigorous science background can annotate the images with only a modest amount of instruction. As such, we designed a custom figure annotation GUI within the MTurk platform and asked workers to draw bounding boxes around each master image in the dataset and then classify them. This allowed us to quickly create a dataset of >3,000 MDI-annotated figures (~18,000 separate images). The basis for training the current version of the figure separator involves augmentation of a random sampling of 2,000 of the annotated images from MTurk and is described in more detail by Jiang and co-authors,[40] as well as in the supplemental notes.

The MCC was used in the discussion of classification results during validation of the figure separator. MCC is computed as follows:

$$\mathrm{MCC} = \frac{TP \times TN - FP \times FN}{\sqrt{(TP+FP) \times (TP+FN) \times (TN+FP) \times (TN+FN)}},$$

where the TP, TN, FP, and FN terminology stands for true positive, true negative, false positive, and false negative, respectively. Note that for each calculation of the MCC for a given class, the multi-class confusion matrix reported (Figure 3) was reduced to a binary matrix based on a "one-vs.-all" scheme.

SUPPLEMENTAL INFORMATION

Supplemental information can be found online at https://doi.org/10.1016/j.patter.2023.100843.

ACKNOWLEDGMENTS

This work is supported by the US Department of Energy, Office of Science, Office of Basic Energy Science, Scientific User Facilities Division Early Career award. We acknowledge support by Laboratory Directed Research and Development (LDRD) funding from the Argonne National Laboratory, provided by the Director, Office of Science, of the US Department of Energy, under contract no. DE-AC02-06CH11357. Work performed at the Center for Nanoscale Materials, a US Department of Energy Office of Science User Facility, was supported by the US DOE, Office of Basic Energy Sciences, under contract no. DE-AC02-06CH11357. We gratefully acknowledge the computing resources provided and operated by the Joint Laboratory for System Evaluation (JLSE) at the Argonne National Laboratory. We gratefully acknowledge the computing resources provided on Bebop, a high-performance computing cluster operated by the Laboratory Computing Resource Center at the Argonne National Laboratory. This research used resources of the National Energy Research Scientific Computing Center, a DOE Office of Science User Facility supported by the Office of Science of the U.S. Department of Energy under Contract No. DE-AC02-05CH11231.

AUTHOR CONTRIBUTIONS

Conceptualization, E.S., W.J., T.S., and M.K.Y.C.; methodology, E.S., W.J., T.S., and M.K.Y.C.; software, E.S., W.J., and T.S.; formal analysis, E.S., W.J., T.S., and M.K.Y.C.; writing – original draft, E.S. and M.K.Y.C.; writing – review & editing, E.S., N.F., O.C., and M.K.Y.C.; funding acquisition, E.S., N.F., and M.K.Y.C.; supervision, O.C. and M.K.Y.C.

DECLARATION OF INTERESTS

The authors declare no competing interests.

Received: October 29, 2022
Revised: January 31, 2023
Accepted: August 21, 2023
Published: October 2, 2023

REFERENCES

1. Howe, B., Lee, P.-s., Grechkin, M., Yang, S.T., and West, J.D. (2017). Deep Mapping of the Visual Literature (IEEE), pp. 1273–1277.
2. Taigman, Y., Yang, M., Ranzato, M.A., and Wolf, L. (2014). Deepface: Closing the Gap to Human-Level Performance in Face Verification (IEEE), pp. 1701–1708.

3. Chen, C., Seff, A., Kornhauser, A., and Xiao, J. (2015). Deepdriving: Learning Affordance for Direct Perception in Autonomous Driving (IEEE), pp. 2722–2730.

4. Silver, D., Huang, A., Maddison, C.J., Guez, A., Sifre, L., Van Den Driessche, G., Schrittwieser, J., Antonoglou, I., Panneershelvam, V., Lanctot, M., et al. (2016). Mastering the game of Go with deep neural networks and tree search. nature *529*, 484–489. https://doi.org/10.1038/nature16961.

5. Sun, C., Shrivastava, A., Singh, S., and Gupta, A. (2017). Revisiting Unreasonable Effectiveness of Data in Deep Learning Era (IEEE), pp. 843–852.

6. Hachtel, J.A., Idrobo, J.C., and Chi, M. (2018). Sub-Ångstrom electric field measurements on a universal detector in a scanning transmission electron microscope. Adv. Struct. Chem. Imaging *4*, 10. https://doi.org/10.1186/s40679-018-0059-4.

7. DeCost, B.L., Hecht, M.D., Francis, T., Webler, B.A., Picard, Y.N., and Holm, E.A. (2017). UHCSDB: ultrahigh carbon steel micrograph database: tools for exploring large heterogeneous microstructure datasets. Integr. Mater. Manuf. Innov. *6*, 197–205. https://doi.org/10.1007/s40192-017-0097-0.

8. Aguiar, J.A., Gong, M.L., Unocic, R.R., Tasdizen, T., and Miller, B.D. (2019). Decoding crystallography from high-resolution electron imaging and diffraction datasets with deep learning. Sci. Adv. *5*, eaaw1949. https://doi.org/10.1126/sciadv.aaw1949.

9. Aversa, R., Modarres, M.H., Cozzini, S., Ciancio, R., and Chiusole, A. (2018). The first annotated set of scanning electron microscopy images for nanoscience. Sci. Data *5*, 180172. https://doi.org/10.1038/sdata.2018.172.

10. Mueller, T., Kusne, A.G., and Ramprasad, R. (2016). Machine learning in materials science: Recent progress and emerging applications. Rev. Comput. Chem. *29*, 186–273. https://doi.org/10.1002/9781119148739.ch4.

11. Kalinin, S.V., Sumpter, B.G., and Archibald, R.K. (2015). Big–deep–smart data in imaging for guiding materials design. Nat. Mater. *14*, 973–980. https://doi.org/10.1038/nmat4395.

12. Kalidindi, S.R., and De Graef, M. (2015). Materials data science: current status and future outlook. Annu. Rev. Mater. Res. *45*, 171–193. https://doi.org/10.1146/annurev-matsci-070214-020844.

13. Jesse, S., Chi, M., Belianinov, A., Beekman, C., Kalinin, S.V., Borisevich, A.Y., and Lupini, A.R. (2016). Big data analytics for scanning transmission electron microscopy ptychography. Sci. Rep. *6*, 26348. https://doi.org/10.1038/srep26348.

14. Schroff, F., Criminisi, A., and Zisserman, A. (2011). Harvesting image databases from the web. IEEE Trans. Pattern Anal. Mach. Intell. *33*, 754–766. https://doi.org/10.1109/TPAMI.2010.133.

15. Li, L.-J., and Fei-Fei, L. (2010). Optimol: automatic online picture collection via incremental model learning. Int. J. Comput. Vis. *88*, 147–168. https://doi.org/10.1007/s11263-009-0265-6.

16. Hua, X.-S., and Li, J. (2015). Prajna: Towards recognizing whatever you want from images without image labeling, p. 1.

17. Yao, Y., Zhang, J., Shen, F., Liu, L., Zhu, F., Zhang, D., and Shen, H.T. (2020). Towards automatic construction of diverse, high-quality image datasets. IEEE Trans. Knowl. Data Eng. *32*, 1199–1211. https://doi.org/10.48550/arXiv.1708.06495.

18. Swain, M.C., and Cole, J.M. (2016). ChemDataExtractor: a toolkit for automated extraction of chemical information from the scientific literature. J. Chem. Inf. Model. *56*, 1894–1904. https://doi.org/10.1021/acs.jcim.6b00207.

19. Kim, E., Huang, K., Tomala, A., Matthews, S., Strubell, E., Saunders, A., McCallum, A., and Olivetti, E. (2017). Machine-learned and codified synthesis parameters of oxide materials. Sci. Data *4*, 170127–170129. https://doi.org/10.1038/sdata.2017.127.

20. Kim, E., Huang, K., Jegelka, S., and Olivetti, E. (2017). Virtual screening of inorganic materials synthesis parameters with deep learning. npj Comput. Mater. *3*, 53. https://doi.org/10.1038/s41524-017-0055-6.

21. Court, C.J., and Cole, J.M. (2018). Auto-generated materials database of Curie and Néel temperatures via semi-supervised relationship extraction. Sci. Data *5*, 180111–180112. https://doi.org/10.1038/sdata.2018.111.

22. Tshitoyan, V., Dagdelen, J., Weston, L., Dunn, A., Rong, Z., Kononova, O., Persson, K.A., Ceder, G., and Jain, A. (2019). Unsupervised word embeddings capture latent knowledge from materials science literature. Nature *571*, 95–98. https://doi.org/10.1038/s41586-019-1335-8.

23. Venugopal, V., Sahoo, S., Zaki, M., Agarwal, M., Gosvami, N.N., and Krishnan, N.M.A. (2021). Looking through glass: Knowledge discovery from materials science literature using natural language processing. Patterns *2*, 100290. https://doi.org/10.1016/j.patter.2021.100290.

24. Mukaddem, K.T., Beard, E.J., Yildirim, B., and Cole, J.M. (2020). ImageDataExtractor: a tool to extract and quantify data from microscopy images. J. Chem. Inf. Model. *60*, 2492–2509. https://doi.org/10.1021/acs.jcim.9b00734.

25. Murphy, R.F., Velliste, M., Yao, J., and Porreca, G. (2001). Searching Online Journals for Fluorescence Microscope Images Depicting Protein Subcellular Location Patterns (IEEE), pp. 119–128.

26. Li, P., Jiang, X., Kambhamettu, C., and Shatkay, H. (2018). Compound image segmentation of published biomedical figures. Bioinformatics *34*, 1192–1199. https://doi.org/10.1093/bioinformatics/btx611.

27. Taschwer, M., and Marques, O. (2018). Automatic separation of compound figures in scientific articles. Multimed. Tools Appl. *77*, 519–548. https://doi.org/10.1007/s11042-016-4237-x.

28. Tsutsui, S., and Crandall, D.J. (2017). A Data Driven Approach for Compound Figure Separation Using Convolutional Neural Networks (IEEE), pp. 533–540.

29. Shi, X., Wu, Y., Cao, H., Burns, G., and Natarajan, P. (2019). Layout-aware Subfigure Decomposition for Complex Figures in the Biomedical Literature (IEEE), pp. 1343–1347.

30. Ahmed, A., Arnold, A., Coelho, L.P., Kangas, J., Sheikh, A.-S., Xing, E., Cohen, W., and Murphy, R.F. (2010). Structured literature image finder: Parsing text and figures in biomedical literature. Web Semant. *8*, 151–154. https://doi.org/10.1016/j.websem.2010.04.002.

31. Park, G., Rayz, J.T., and Pouchard, L. (2020). Figure Descriptive Text Extraction Using Ontological Representation.

32. Agarwal, S., and Yu, H. (2009). FigSum: Automatically Generating Structured Text Summaries for Figures in Biomedical Literature (American Medical Informatics Association), p. 6.

33. Venugopal, V., Broderick, S.R., and Rajan, K. (2019). A picture is worth a thousand words: applying natural language processing tools for creating a quantum materials database map. MRS Communications *9*, 1134–1141. https://doi.org/10.1557/mrc.2019.136.

34. Reitz, K. (2020). Requests (Python). http for humans.

35. Honnibal, M., and Montani, I. (2017). *spaCy 2: Natural language understanding with Bloom embeddings, convolutional neural networks and incremental parsing*. spaCy: Industrial-Strength NLP.

36. Beltagy, I., Lo, K., and Cohan, A. (2019). SciBERT: A pretrained language model for scientific text. Preprint at arXiv. https://doi.org/10.48550/arXiv.1903.10676.

37. Cruse, K., Trewartha, A., Lee, S., Wang, Z., Huo, H., He, T., Kononova, O., Jain, A., and Ceder, G. (2022). Text-mined dataset of gold nanoparticle synthesis procedures, morphologies, and size entities. Sci. Data *9*, 234. https://doi.org/10.1038/s41597-022-01321-6.

38. Gupta, T., Zaki, M., Krishnan, N.M.A., and Mausam. (2022). MatSciBERT: A materials domain language model for text mining and information extraction. npj Comput. Mater. *8*, 102. https://doi.org/10.1038/s41524-022-00784-w.

39. Material Science Characterization Keywords. https://github.com/MaterialEyes/exsclaim/blob/dev/exsclaim/captions/models/characterization.yml.

40. Jiang, W., Schwenker, E., Spreadbury, T., Ferrier, N., Chan, M.K., and Cossairt, O. (2021). A Two-Stage Framework for Compound Figure Separation (IEEE), pp. 1204–1208.

41. Jiang, W., Li, K., Spreadbury, T., Schwenker, E., Cossairt, O., and Chan, M.K.Y. (2022). Plot2Spectra: an automatic spectra extraction tool. Digital Discovery *1*, 719–731. https://doi.org/10.1039/D1DD00036E.

CellPress
OPEN ACCESS

42. Shi, Q., Si, K.J., Sikdar, D., Yap, L.W., Premaratne, M., and Cheng, W. (2016). Two-dimensional bipyramid plasmonic nanoparticle liquid crystalline superstructure with four distinct orientational packing orders. ACS Nano *10*, 967–976. https://doi.org/10.1021/acsnano.5b06206.

43. Redmon, J., and Farhadi, A. (2018). Yolov3: An incremental improvement. Preprint at arXiv. https://doi.org/10.48550/arXiv.1804.02767.

44. He, K., Zhang, X., Ren, S., and Sun, J. (2016). Deep Residual Learning for Image Recognition (IEEE), pp. 770–778.

45. Schwenker, E., Jiang, W., Spreadbury, T., Ferrier, N., Cossairt, O., and Chan, M.K.Y. (2021). EXSCLAIM! Validation Dataset - Selections from Amazon Mechanical Turk Benchmark.

46. Blaiszik, B., Chard, K., Pruyne, J., Ananthakrishnan, R., Tuecke, S., and Foster, I. (2016). The materials data facility: data services to advance materials science research. Jom *68*, 2045–2052. https://doi.org/10.1007/s11837-016-2001-3.

47. Blaiszik, B., Ward, L., Schwarting, M., Gaff, J., Chard, R., Pike, D., Chard, K., and Foster, I. (2019). A data ecosystem to support machine learning in materials science. MRS Communications *9*, 1125–1133. https://doi.org/10.1557/mrc.2019.118.

48. Willhammar, T., Sentosun, K., Mourdikoudis, S., Goris, B., Kurttepeli, M., Bercx, M., Lamoen, D., Partoens, B., Pastoriza-Santos, I., Pérez-Juste, J., et al. (2017). Structure and vacancy distribution in copper telluride nanoparticles influence plasmonic activity in the near-infrared. Nat. Commun. *8*, 14925. https://doi.org/10.1038/ncomms14925.

49. Zhao, Y., Kuai, L., Liu, Y., Wang, P., Arandiyan, H., Cao, S., Zhang, J., Li, F., Wang, Q., Geng, B., and Sun, H. (2015). Well-constructed single-layer molybdenum disulfide nanorose cross-linked by three dimensional-reduced graphene oxide network for superior water splitting and lithium storage property. Sci. Rep. *5*, 8722. https://doi.org/10.1038/srep08722.

50. Hofer, C., Skákalová, V., Görlich, T., Tripathi, M., Mittelberger, A., Mangler, C., Monazam, M.R.A., Susi, T., Kotakoski, J., and Meyer, J.C. (2019). Direct imaging of light-element impurities in graphene reveals triple-coordinated oxygen. Nat. Commun. *10*, 4570. https://doi.org/10.1038/s41467-019-12537-3.

51. Matthews, B.W. (1975). Comparison of the predicted and observed secondary structure of T4 phage lysozyme. Biochim. Biophys. Acta *405*, 442–451. https://doi.org/10.1016/0005-2795(75)90109-9.

52. Schwenker, E., Jiang, W., Spreadbury, T., Ferrier, N., Cossairt, O., and Chan, M.K.Y. (2021). EXSCLAIM! Exploratory Dataset - Nanostructure Images from Nature Journals.

53. Mastria, R., Scarfiello, R., Altamura, D., Giannini, C., Liscio, A., Kovtun, A., Bianco, G.V., Bruno, G., Grillo, V., Tavabi, A.H., et al. (2019). In-plane aligned colloidal 2D WS2 nanoflakes for solution-processable thin films with high planar conductivity. Sci. Rep. *9*, 9002. https://doi.org/10.1038/s41598-019-45192-1.

54. Wang, Y.-X., Yang, J., Chou, S.-L., Liu, H.K., Zhang, W.-x., Zhao, D., and Dou, S.X. (2015). Uniform yolk-shell iron sulfide–carbon nanospheres for superior sodium–iron sulfide batteries. Nat. Commun. *6*, 8689. https://doi.org/10.1038/ncomms9689.

55. Tripathi, R.M., and Chung, S.J. (2020). Reclamation of hexavalent chromium using catalytic activity of highly recyclable biogenic Pd (0) nanoparticles. Sci. Rep. *10*, 640. https://doi.org/10.1038/s41598-020-57548-z.

56. Pujala, R.K., Pujala, R.K., and Dhara, S. (2019). Colloidal analogues of polymer chains, ribbons and 2D crystals employing orientations and interactions of nano-rods dispersed in a nematic liquid crystal. Sci. Rep. *9*, 4652. https://doi.org/10.1038/s41598-019-40198-1.

57. Wang, Z., Ju, Y., Ali, Z., Yin, H., Sheng, F., Lin, J., Wang, B., and Hou, Y. (2019). Near-infrared light and tumor microenvironment dual responsive size-switchable nanocapsules for multimodal tumor theranostics. Nat. Commun. *10*, 4418. https://doi.org/10.1038/s41467-019-12142-4.

58. Zhuang, Z., Wang, Y., Xu, C.-Q., Liu, S., Chen, C., Peng, Q., Zhuang, Z., Xiao, H., Pan, Y., Lu, S., et al. (2019). Three-dimensional open nano-netcage electrocatalysts for efficient pH-universal overall water splitting. Nat. Commun. *10*, 4875. https://doi.org/10.1038/s41467-019-12885-0.

59. Hossain, R., Pahlevani, F., and Sahajwalla, V. (2020). Revealing the mechanism of extraordinary hardness without compensating the toughness in a low alloyed high carbon steel. Sci. Rep. *10*, 181. https://doi.org/10.1038/s41598-019-55803-6.

60. Hong, C.-H., Oh, S.K., Kim, T.K., Cha, Y.-J., Kwak, J.S., Shin, J.-H., Ju, B.-K., and Cheong, W.-S. (2015). Electron beam irradiated silver nanowires for a highly transparent heater. Sci. Rep. *5*, 17716. https://doi.org/10.1038/srep17716.

61. Huang, H., Polavarapu, L., Sichert, J.A., Susha, A.S., Urban, A.S., and Rogach, A.L. (2016). Colloidal lead halide perovskite nanocrystals: synthesis, optical properties and applications. NPG Asia Mater. *8*, e328. https://doi.org/10.1038/am.2016.167.

62. Kasukabe, T., Nishihara, H., Kimura, K., Matsumoto, T., Kobayashi, H., Okai, M., and Kyotani, T. (2017). Beads-milling of waste Si sawdust into high-performance nanoflakes for lithium-ion batteries. Sci. Rep. *7*, 42734. https://doi.org/10.1038/srep42734.

63. Devlin, J., Chang, M.-W., Lee, K., and Toutanova, K. (2018). Bert: Pre-training of deep bidirectional transformers for language understanding. Preprint at arXiv. https://doi.org/10.48550/arXiv.1810.04805.

64. Yang, Z., Dai, Z., Yang, Y., Carbonell, J., Salakhutdinov, R.R., and Le, Q.V. (2019). Xlnet: Generalized autoregressive pretraining for language understanding. Adv. Neural Inf. Process. Syst. *32*. https://doi.org/10.48550/arXiv.1906.08237.

65. Brown, T., Mann, B., Ryder, N., Subbiah, M., Kaplan, J.D., Dhariwal, P., Neelakantan, A., Shyam, P., Sastry, G., and Askell, A. (2020). Language models are few-shot learners. Adv. Neural Inf. Process. Syst. *33*, 1877–1901. https://doi.org/10.48550/arXiv.2005.14165.

66. Ren, S., He, K., Girshick, R., and Sun, J. (2015). Faster r-cnn: Towards real-time object detection with region proposal networks. Adv. Neural Inf. Process. Syst. *28*. https://doi.org/10.48550/arXiv.1506.01497.

67. Zuo, Z., Shuai, B., Wang, G., Liu, X., Wang, X., Wang, B., and Chen, Y. (2015). Convolutional Recurrent Neural Networks: Learning Spatial Dependencies for Image Representation (IEEE), pp. 18–26.

Patterns, Volume *4*

Supplemental information

EXSCLAIM!: Harnessing materials science literature for self-labeled microscopy datasets

Eric Schwenker, Weixin Jiang, Trevor Spreadbury, Nicola Ferrier, Oliver Cossairt, and Maria K.Y. Chan

Supplemental Notes

Introduction

The sections outlined in this document provide further details on results and experimental procedures, in support of the conclusions made in the corresponding sections of the main paper.

Results

Journal Scraper

The reason that the default parsers are designed for Nature, ACS, and RSC families relates to both impact and the fact that their contents are served in fundamentally different ways. Nature and ACS have static content, while RSC's content is served dynamically. Consequently, with this collection of parsers, we cover two common ways information could be obtained from a website, which leaves this tool open to be easily extended to any open source journal. All figure/caption extraction is performed directly from the HTML article and does not require PDF downloads. This is a design choice based on the observation that most open-source content is available directly from the HTML text and the ability to download the PDF is a convenience rather than a necessity.

Figure Separator

The *figure separator* comprises two primary modules:

1. Subfigure Label Detector (YOLOv3 + ResNet152)

   YOLOv3 is trained for label detection and ResNet152 is trained for label recognition. For the YOLOv3 implementation, refer to: https://github.com/WeixinGithubJiang/PyTorch_YOLOv3

YOLOv3:

```
MODEL:
  TYPE: YOLOv3
  BACKBONE: darknet53
  ANCHORS: [[6, 7], [9, 10], [10, 14],
            [13, 11], [16, 15], [15, 20],
            [21, 19], [24, 24], [34, 31]]
  ANCH_MASK: [[6, 7, 8], [3, 4, 5], [0, 1, 2]]
  N_CLASSES: 15
TRAIN:
  LR: 0.001
  MOMENTUM: 0.9
  DECAY: 0.0005
  BURN_IN: 1000
  MAXITER: 20000
  STEPS: (400000, 450000)
  BATCHSIZE: 4
  SUBDIVISION: 16
  IMGSIZE: 608
  LOSSTYPE: l2
  IGNORETHRE: 0.7
AUGMENTATION:
  RANDRESIZE: True
  JITTER: 0.3
  RANDOM_PLACING: True
  HUE: 0.1
  SATURATION: 1.5
  EXPOSURE: 1.5
  LRFLIP: False
  RANDOM_DISTORT: True
TEST:
  CONFTHRE: 0.01
  NMSTHRE: 0.1
  IMGSIZE: 608
NUM_GPUS: 1
GPU_ID: 0
```

ResNet152:

We used a pre-trained model from ImageNet as weight initialization, then trained with a mixture of real MTurk-labeled images and synthetic. The Adam optimizer was used with initial training weights 0.001 and decay every 10000 iteration. A seed was not used.

2. Subfigure detector (YOLOv3)

Model details are similar to subfigure label detection, however, the config is a bit different (KMeans was used to find the proper anchor boxes).

YOLOv3:

```
MODEL:
  TYPE: YOLOv3
  BACKBONE: darknet53
  ANCHORS: [[185, 147], [94, 87], [133, 126],
            [102, 212], [234, 102], [204, 199],
            [390, 123], [177, 368], [318, 230]]
  ANCH_MASK: [[6, 7, 8], [3, 4, 5], [0, 1, 2]]
  N_CLASSES: 15
TRAIN:
  LR: 0.001
  MOMENTUM: 0.9
  DECAY: 0.0005
  BURN_IN: 1000
  MAXITER: 50000
  STEPS: (400000, 450000)
  BATCHSIZE: 4
  SUBDIVISION: 16
  IMGSIZE: 608
  LOSSTYPE: l2
  IGNORETHRE: 0.7
AUGMENTATION:
  RANDRESIZE: True
  JITTER: 0.3
  RANDOM_PLACING: True
  HUE: 0.1
  SATURATION: 1.5
  EXPOSURE: 1.5
  LRFLIP: False
  RANDOM_DISTORT: True
TEST:
  CONFTHRE: 0.1
  NMSTHRE: 0.1
  IMGSIZE: 416
NUM_GPUS: 1
GPU_ID: 0
```

Both YOLOv3 models were trained on ≈800 figures with SGD. Testing comprised 1164 images without a dedicated validation set (some further details are included in the ICIP paper https://arxiv.org/pdf/2101.09903.pdf). For ResNet152, training is on-the-fly because the synthesized images are re-generated every epoch. All models are trained on 2 TITAN-X GPUs, each one with 12GB memory.

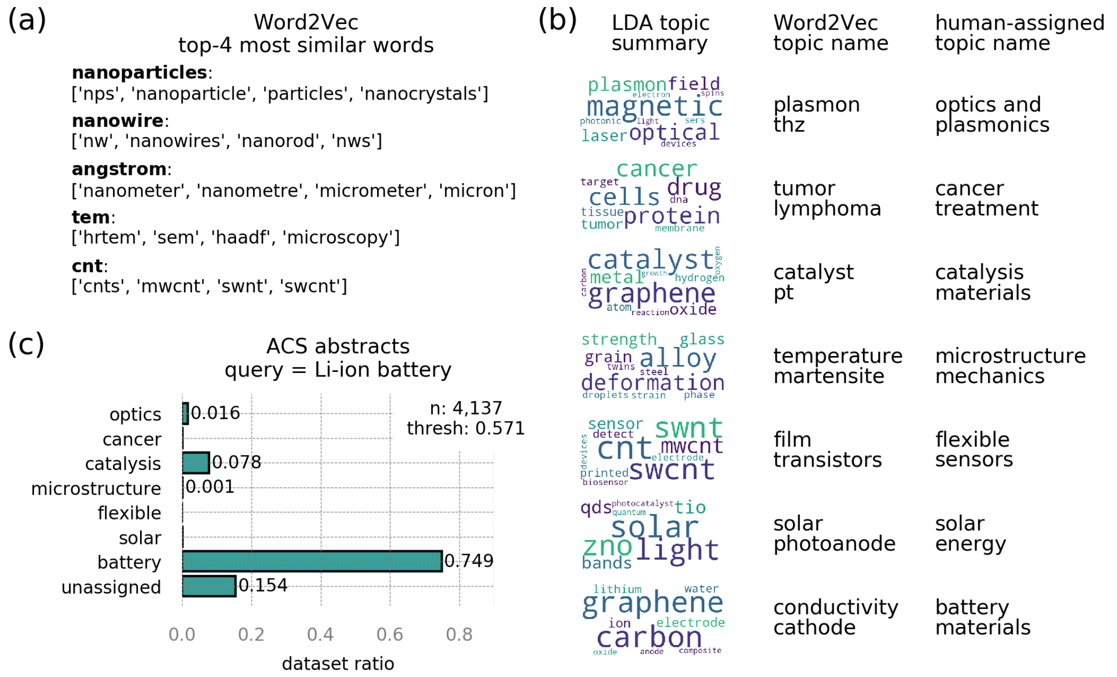

Figure S1. **Word Embeddings and Topic Modeling Highlights.** (a) Word embedding examples for a Word2Vec model trained on abstract and introduction texts from the nanostructure query. (b) LDA topic modeling applied to the introduction and abstract texts reveals some of the most popular technological applications of nanostructures. The Word2Vec topic name and human-assigned topic name represent further attempts to summarize the words of the topics into more concise titles. (c) Distribution of topics assigned to a group of 4237 abstracts collected from a query of American Chemical Society (ACS) journals for Li-ion batteries.

Annotation Expansion with NLP

The approach for determining topics and "semantic word similarity" in the main paper, uses two popular techniques from NLP: word embeddings and statistical topic modeling. These concepts are further explained below.

1. Word Embeddings (Word2Vec)

EXSCLAIM! leverages the popular unsupervised Word2Vec technique[1] to learn high quality vectors (embeddings) for words associated with the captions returned in the nanostructure query. To demonstrate how Word2Vec can be used for word associations, Figure S1a highlights simple "closest words" lookup examples. Without being explicitly associated, "nanoparticles" and "nanowire" are placed in close proximity to their abbreviations, "nps" and "nw", respectively. Additionally, 3D "nanoparticles" are closely associated with another 3D nanostructure, such as a "nanocrystal", and "nanowire" is placed near a similar 2D "nanorod". The "angstrom" unit of length is placed closely to "nanometer" and "micrometer", and interestingly, scale is even preserved in the ordering (*i.e.,* angstrom is closer in scale to a nm than μm). These sorts of quantitative relationships are not uncommon and are described in further detail in the original paper.[1] The `gensim` Python library (https://radimrehurek.com/gensim/) was used to train a Word2Vec model on 26k abstract and introduction texts scraped from nanoscience journals, with the following parameters: `min_count = 25, size = 200, iter = 500`.

2. Statistical Topic Modeling (Latent Dirichlet Analysis)

When all abstract and introductory texts related to the general nanostructures *search query* are considered together, certain topics (groups of related words) arise from high-frequency word occurrences and common word orderings. The word clouds in Figure S1b illustrate how Latent Dirichlet Analysis (LDA),[2] a popular technique used for topic modeling, provides an unsupervised clustering of related words, (from the nanostructure query) into topics. Unfortunately, LDA does not provide a topic name to the words it clusters together. To this end, we illustrate how Word2Vec can be leveraged to create topic names, and show how they align with rational human-suggested titles. For example, the topic containing "catalyst", "metal", "graphene", "reaction", etc. is given the Word2Vec topic name of "catalyst", "pt", which is easily understood to represent the general class of "catalysis materials". To demonstrate that the LDA has learned topics in an appropriate fashion, we collected 4137 abstract and introduction texts from a *search query* of ACS journal family for "Li-ion batteries", and in Figure S1c, we observe that the majority of the documents were categorized explicitly as belonging to batteries, or the highly related/overlapping catalysis category.

For LDA topic modeling, abstract and introduction texts were transformed into a corpus of TFIDF vectors and LdaMulticore was used for training with `num_topics=7 ,passes=64 ,workers=4`. The number of topics was selected based on its coherence score (`u_mass`). The coherence score can be used to assess the quality of the learned topics for a given number of possible topics. While the specific intent of the original paper describing the `u_mass` evaluation metric[3] was to develop methods to create large numbers of high-quality topics from domain-specific corpora, for purposes of demonstrating the potential utility of unsupervised topic models in context of the EXSCLAIM! pipeline, we restrict our analysis of topic coherence to a relatively small number of topics (5 - 10). This allows the reader to make a simple quality judgment, and is a generally accepted approach (*i.e.,* including a small number biases selection against the potential for *very poor* topic selection). For the specific `u_mass` metric, numbers closer to zero indicate higher coherence, as the measure itself is the log ratio of a document frequency of specific word type pairs to a document frequency of a specific word type.[3] The following table shows each topic number with its corresponding `u_mass` value, and reveals in this case that the choice of "7" topics has the best coherence score.

Topics	Coherence (`u_mass`)
5	-3.143
6	-4.354
**7**	**-1.857**
8	-4.792
9	-5.429
10	-6.122

Experimental Procedures

Custom Regex Matching

(a)

Caption Text (Raw) Text adapted from: *Inorg. Chem. Front.*, 2019, **6**, 1382-1387

(a) and **(b)** TEM images of 1.93 wt% Ru–WSe2. **(c)** HRTEM image of 1.93 wt% Ru–WSe2. **(d, e)** The enlarged area denoted in **(c)** corresponds to the HRTEM images of WSe2. **(f)** HAADF-STEM image of 1.93 wt% Ru–WSe2. **(g - i)** The EDS mapping of Ru, W, and Se, respectively.

(b)

Subfigure Token	Token Class	Subfigures Implied
(a) and (b)	parenthesis_02_and_alpha_02	[a , b]
(c)	parenthesis_02_none_alpha_01	[c]
(d, e)	parenthesis_02_comma_alpha_02	[d , e]
(c)	parenthesis_02_none_alpha_01	[c]
(f)	parenthesis_02_none_alpha_01	[f]
(g – i)	parenthesis_02_dash_alpha_03	[g , h , i]

(c)

Caption Text (Custom POS Tagging)

(‘(a) and (b)’, ‘CAP’),(‘TEM images’, ‘NC’),(‘of’, ‘IN’),(‘1.93 wt\% Ru–WSe2’, ‘NC’), … ,(‘(d, e)’, ‘CAP’),
(‘The enlarged area’, ‘NC’),(‘denoted’, ‘IR’),(‘in’, ‘IN’),(‘(c)’, ‘CAP’),(‘corresponds’, ‘IR’),(‘to’, ‘IN’) …

(d)

Pattern #1	Interpretation	
(“CAP”, “!”, “NC”, “IN”, “*”, “.”)	“CAP” -	Start with a caption delimiter.
	“!” -	Do not record any text until …
	“NC” -	the next noun chunk that contains a …
	“IN” -	preposition immediately after.
	“*” -	Continue including all text until …
	“.” -	a full stop is detected.

(e)

Assigned Text From Pattern #1 (a and b), (c), …

(a) TEM images of 1.93 wt% Ru–WSe2 | **(b)** TEM images of 1.93 wt% Ru–WSe2 | **(c)** HRTEM image of 1.93 wt% Ru–WSe2 …

Figure S2. **Demonstration of Custom Regex Methodology on Example Caption.** (a) The raw caption text. (b) Examples of *subfigure token* classifications. (c) Result of custom POS tagging. (d) Example sentence-level regex pattern and plain English interpretation. (e) Examples of the final assigned text for the selected subfigures.

Image Scaling Estimation

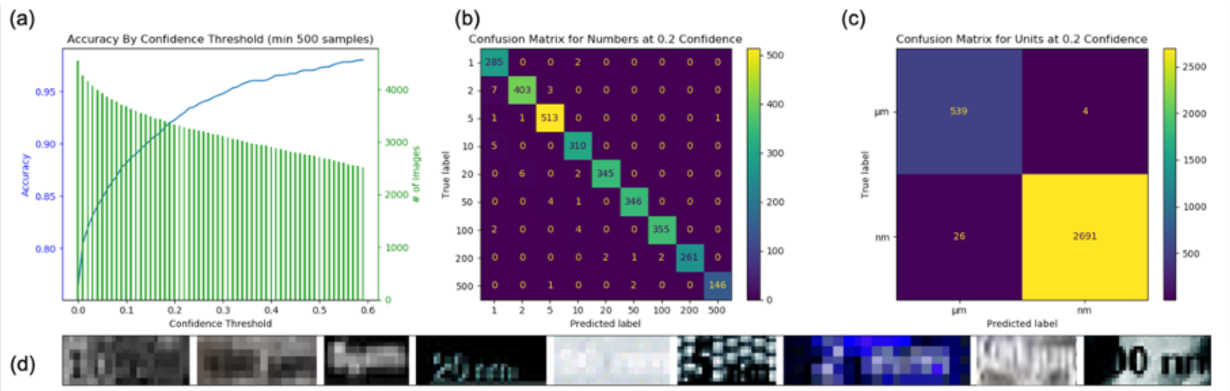

Figure S3. **Image Scaling Estimation Accuracy Examples.** (a) Estimation accuracy as a function of confidence threshold, including the number of images present at a given threshold. For all thresholds shown, there are at least 500 samples. When the confidence level associated with the scale bar label detection is ≈0.6, the overall accuracy (percentage of scale bar labels that are exactly right – both the number the unit) is > 0.95. (b, c) The confusion matrices highlight the accuracy of the predicted number and unit components for recognition of the scale bar label. Larger scales (*i.e.*, mm and cm) were not adequately represented in the training set, so they are not part of the test set. Both number and scale recognition accuracy is high at the 0.2 threshold (≈92% and ≈99% respectively for the labels shown). (d) Common instances of low-resolution and low-contrast conditions that are responsible for a majority of the prediction errors.

The image scaling estimation comprises two primary modules:

1. Scale Bar Detector (Faster RCNN with a Resnet-50-FPN backbone)

The *scale bar detector* was trained on the same dataset as the *figure separator*, with the objective of detecting scale bar lines and scale bar labels. A Gaussian blur with a 5×5 kernel size was randomly applied (with probability of .25) to training images. The Faster RCNN was pretrained on COCO train2017 and the backbone was pretrained on Imagenet. The Adam optimizer was used with a learning rate of 0.001, and a batch size of 32, and run for about 200 epochs. The non-maximum suppression method was used for inference. Figures were split between the test and train set by paper (all figures from a given paper will be in the same set to avoid leakage). The models were trained on a 4×NVIDIA Tesla V100 SXM2 with 32GB HBM2 and took about 4 minutes per epoch.

2. Scale Label Reader (Convolutional Recurrent Neural Network)

The CNN portion had 3 input channels (RGB) of 128×512 pixels, 22 output channels, and a sequence length of 32 and 6 layers with 64, 64, 64, 128, 128, and 128 output channels. This was fed into a bi-directional LSTM and then finally a fully connected output layer. The output was fed through LogSoftmax. CTC loss was used. Training proceeded for ≈1000 epochs with a batch size of 16. The model was trained and tested on a 4×NVIDIA Tesla V100 SXM2 with 32GB HBM2 with synthetic data. The synthetic data was generated by randomly placing random strings of 3-8 characters from the potential output channels on a set of textless microscopy images. Reported metrics were based on performance on the MTurk *figure separator* dataset, and took about 1 minute per epoch.

References

1. Mikolov, T., Sutskever, I., Chen, K., Corrado, G.S., and Dean, J. (2013). Distributed representations of words and phrases and their compositionality. Advances in neural information processing systems 26. 10.48550/arXiv.1310.4546.

2. Blei, D.M., Ng, A.Y., and Jordan, M.I. (2003). Latent dirichlet allocation. Journal of machine Learning research 3, 993-1022.

3. Mimno, D., Wallach, H., Talley, E., Leenders, M., and McCallum, A. (2011). Optimizing semantic coherence in topic models. pp. 262-272.
